# Supplementary material for: Synaptogenic gene therapy with FGF22 improves circuit plasticity and functional recovery following spinal cord injury
Source: EMBO Mol Med. 2023 Jan 5;15(2):e16111. doi: 10.15252/emmm.202216111 (PMC9906383; doi:10.15252/emmm.202216111)
Supplement: Supplementary file 1 — Expanded View Figures PDF [file EMMM-15-e16111-s001.pdf]

Expanded View Figures

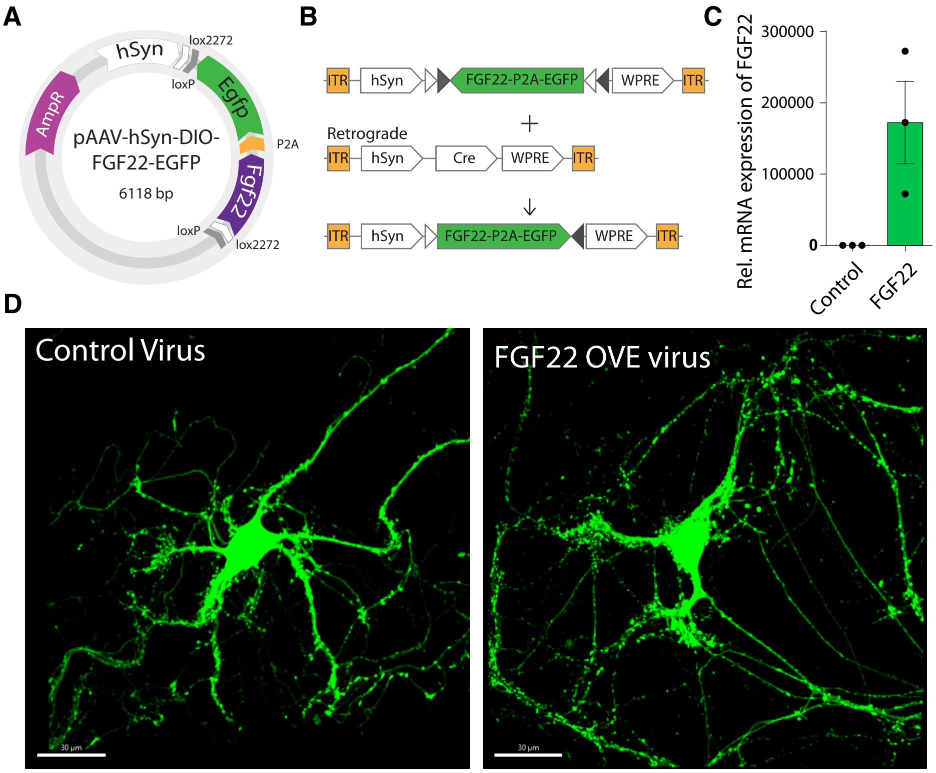

Figure EV1. Testing viral constructs in primary cortical neurons.

A, B Illustration of plasmid construct and double inverted cre-lox system for conditional FGF22 overexpression.

C qPCR quantification of Fgf22 overexpression in cultured HEK cells ($n = 3$ replicates per condition). Data are expressed as mean ± SEM.

D Representative confocal images of *in vitro* cortical neurons from vGlut2-cre mice (green) infected with (left) rAAV-hSyn-DIO-EGFP and (right) with rAAV-hSyn-DIO-FGF22-EGDP. Scale bar equals 20 µm.

© 2023 The Authors

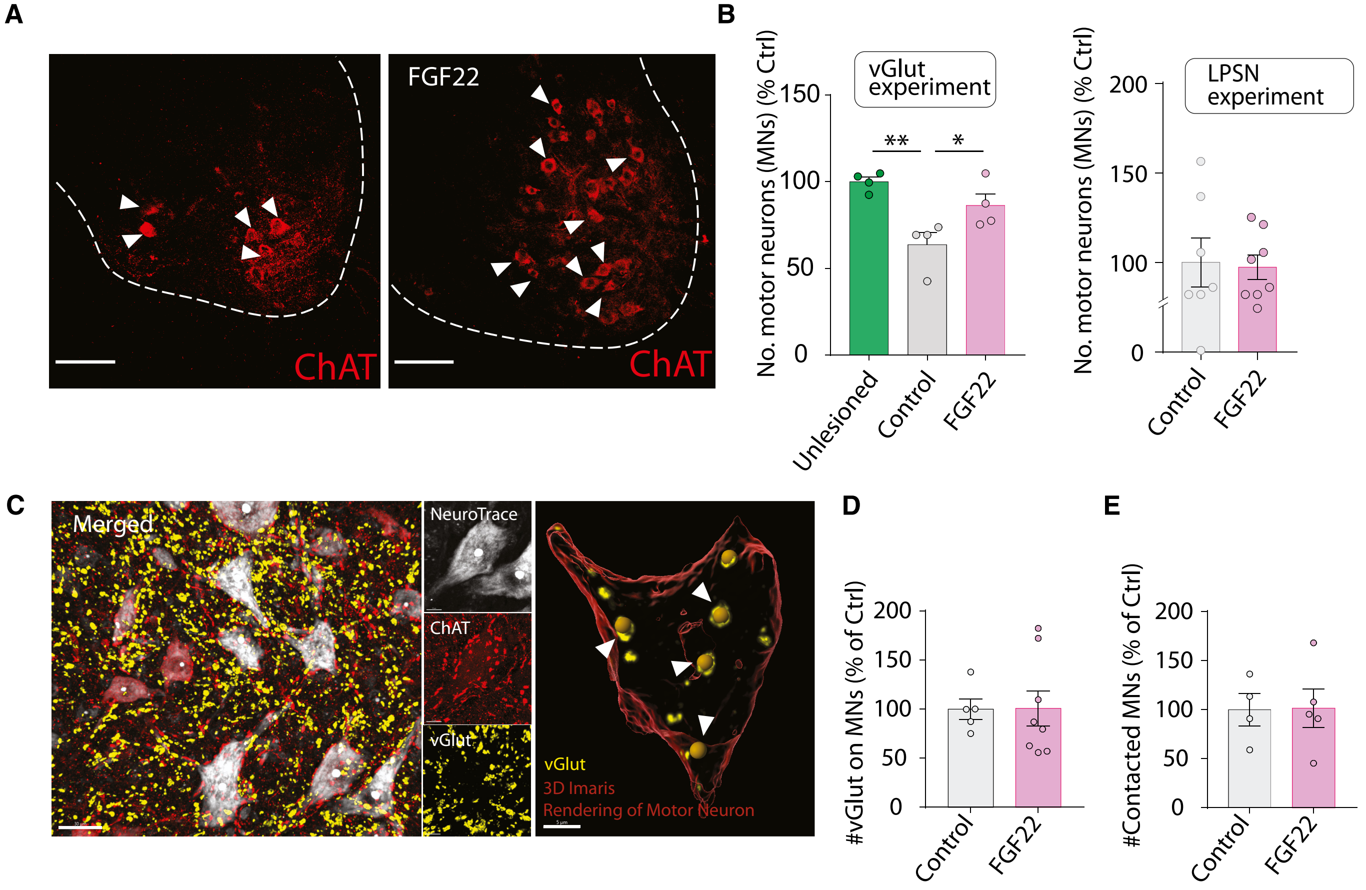

Figure EV2. FGF22 triggers motoneuron survival when delivered to vGlut-cre mice.

A Representative confocal images of ChAT-based motoneuron immunostaining (red).

B Quantification of motoneuron survival following FGF22 overexpression in excitatory neurons (left) and LPSN (right) (4–8 mice per group; green: unlesioned mice; gray: lesioned mice treated with control virus and magenta: lesioned mice treated with FGF22 virus).

C (Left) Representative confocal images of motoneurons (red: ChAT staining and white: Neurotrace 435) and vGlut staining (yellow). (Right) 3D surface rendered in Imaris Software, showing quantification of vGlut puncta on a motor neuron.

D Quantification of vGlut spots onto motoneurons, normalized to neuron surface area in LPSN experiment. Results are represented as percentage of control (n = 5–8 animals per group).

E Quantification of vGlut projections onto motoneurons. Results are represented as percentage of control (n = 4–5 per group).

Data information: $^{*}P < 0.05$ and $^{**}P < 0.01$. Unpaired t-test for panel (B) (unlesioned vs. control) and Mann–Whitney test for panel (B) (control vs. FGF22). Unpaired t-test for panel (B) (LPSN experiment) and panels (D) and (E). Scale bar for (A) equals 50 µm and for (C) left 30 µm and for (C) right 5 µm. Arrowheads in (A) represent $ChAT^{+}$ motoneurons. Arrowheads in (C) represent vGlut contact onto motoneuron. Data are expressed as mean ± SEM.

© 2023 The Authors

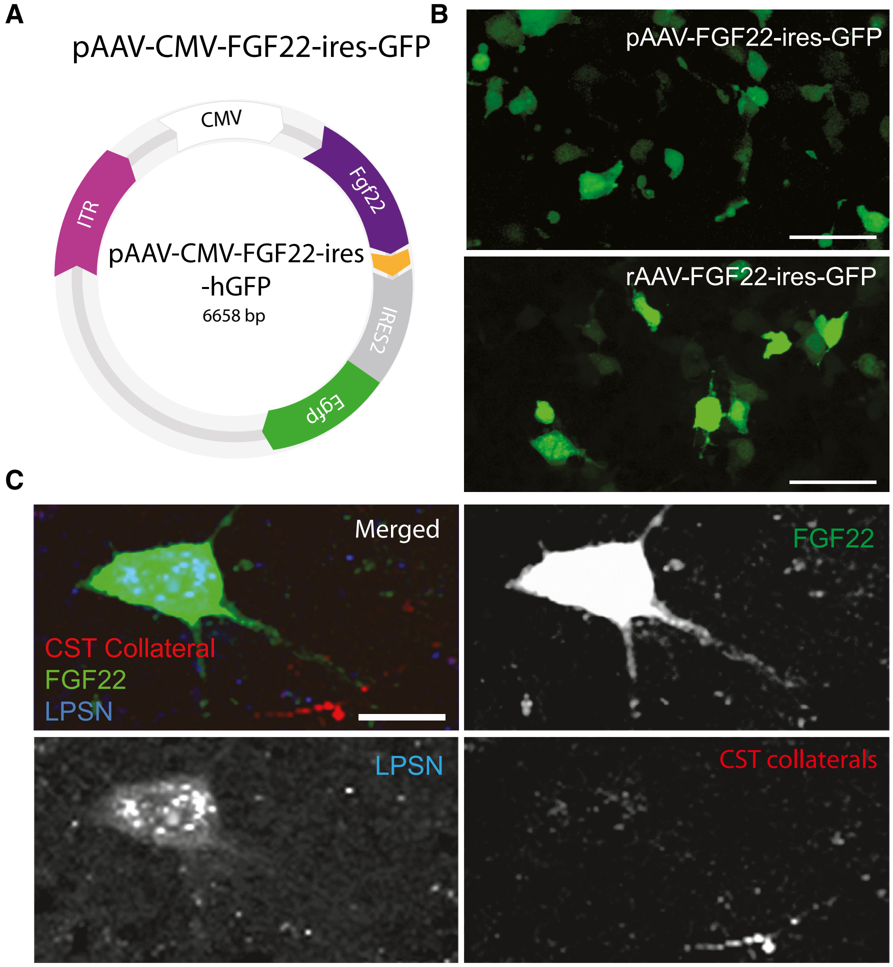

Figure EV3. Design and validation of plasmid used for testing therapeutic potentials.

A Illustration of plasmid construct used for therapeutic treatment.
B Representative images of plasmids tested in HEK cells.
C Representative confocal images of long propriospinal neuron retrogradely labeled with FluoroGold (blue), FGF22 overexpression (green) and CST collateral (white) contacting FGF22 overexpressing neuron.

Data information: Scale bar in (B) equals ~ 30 μm and (C) equals 5 μm.

 © 2023 The Authors

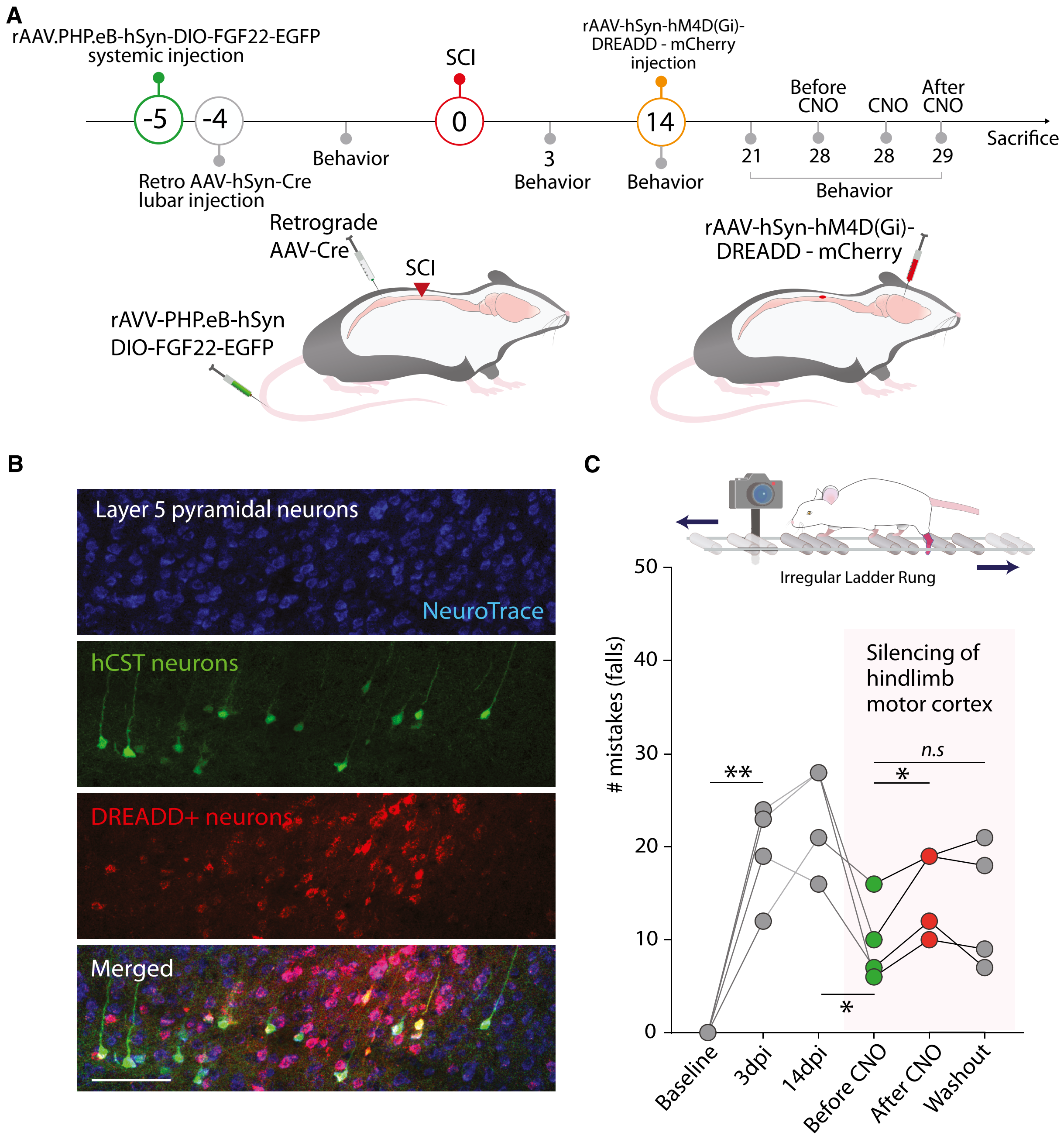

Figure EV4. Chemogenetic silencing of the hindlimb motor cortex demonstrates its contribution to functional recovery following spinal cord injury.

A Time line of the experiment in which FGF22 is overexpressed in long propriospinal neurons and silencing DREADDs are delivered to the hindlimb motor cortex.

B Confocal images of the DREADDs expressing neurons retrogradely labeled with AAV-Cre (Neurotrace: blue; Retrogradely labeled neurons: green; DREADD+ neurons: red). Scale bar equals 100 μm.

C Longitudinal quantifications of mistakes in the irregular ladder rung at baseline, 3-day postinjury (dpi), 14 dpi, 28 dpi before CNO administration (green dots), 28 dpi 30 min after CNO administration (red dots) and 29 dpi (CNO washout; gray dots) and scheme of the ladder rung task.

Data information. ns: $P > 0.05$; *$P < 0.05$ and **$P < 0.01$. For general group difference one-way repeated-measures ANOVA was used. In order to assess recovery of individual mice paired *t*-test was used (panel C).

© 2023 The Authors
