# Supplementary material for: Synaptogenic gene therapy with FGF22 improves circuit plasticity and functional recovery following spinal cord injury
Source: EMBO Mol Med. 2023 Jan 5;15(2):e16111. doi: 10.15252/emmm.202216111 (PMC9906383; doi:10.15252/emmm.202216111)
Supplement: Supplementary file 2 — PDF+ [file EMMM-15-e16111-s003.pdf]

Article

TRANSPARENT PROCESS

OPEN ACCESS

EMBO Molecular Medicine

Synaptogenic gene therapy with FGF22 improves circuit plasticity and functional recovery following spinal cord injury

Almir Aljović[1,2,3,†], Anne Jacobi[1,2,†,‡], Maite Marcantoni[1,2], Fritz Kagerer[1,2,4], Kristina Loy[1,2], Arek Kendirli[1,2,3], Jonas Bräutigam[1,2], Luca Fabbio[1,2], Valérie Van Steenbergen[1,2], Katarzyna Pleśniar[1,2], Martin Kerschensteiner[1,2,5] & Florence M Bareyre[1,2,5,*]

Abstract

Functional recovery following incomplete spinal cord injury (SCI) depends on the rewiring of motor circuits during which supraspinal connections form new contacts onto spinal relay neurons. We have recently identified a critical role of the presynaptic organizer FGF22 for the formation of new synapses in the remodeling spinal cord. Here, we now explore whether and how targeted overexpression of FGF22 can be used to mitigate the severe functional consequences of SCI. By targeting FGF22 expression to either long propriospinal neurons, excitatory interneurons, or a broader population of interneurons, we establish that FGF22 can enhance neuronal rewiring both in a circuit-specific and comprehensive way. We can further demonstrate that the latter approach can restore functional recovery when applied either on the day of the lesion or within 24 h. Our study thus establishes viral gene transfer of FGF22 as a new synaptogenic treatment for SCI and defines a critical therapeutic window for its application.

Keywords adeno-associated virus; FGF22; gene therapy; recovery; spinal cord injury

Subject Categories Genetics, Gene Therapy & Genetic Disease; Neuroscience

DOI 10.15252/emmm.202216111 | Received 1 April 2022 | Revised 14 December 2022 | Accepted 15 December 2022 | Published online 5 January 2023

EMBO Mol Med (2023) 15: e16111

Introduction

Circuit rewiring following incomplete spinal cord injury is an important component of functional recovery (Bareyre *et al*, 2004; Girgis *et al*, 2007; Van Den Brand *et al*, 2012; Bradley *et al*, 2019; Engmann *et al*, 2020; Brommer *et al*, 2021). One example of such plasticity is the ability for hindlimb corticospinal projection pathways to respond to incomplete thoracic lesions by the *de novo* formation of intraspinal detour circuits that bypass the lesion site and reconnect the upper corticospinal projection neurons to the lumbar spinal cord (Bareyre *et al*, 2004; Lang *et al*, 2012). A critical step in this remodeling process is the formation of new synaptic contacts in the cervical spinal cord between supraspinal projections, in this case the hindlimb corticospinal tract (hCST), and spinal relay interneurons, in particular long propriospinal neurons. The functional significance of such intraspinal detour circuits is well-established (Bareyre *et al*, 2004; Courtine *et al*, 2008; Van Den Brand *et al*, 2012; Jacobi *et al*, 2015; Loy *et al*, 2018; Bradley *et al*, 2019; Brommer *et al*, 2021), and the mechanisms that regulate their formation are thus of high scientific and biomedical interest.

FGF22, a target-derived presynaptic organizer that is critical for the establishment of new excitatory synapses during development (Umemori *et al*, 2004; Terauchi *et al*, 2010; Dabrowski *et al*, 2015) and the organization of synapses in adulthood (Pasaoglu & Schikorski, 2016; Li *et al*, 2020), has emerged as an important endogenous contributor to detour circuit formation. We could previously show that FGF22 and its receptors FGFR1 and FGFR2 remain constitutively expressed in the adult spinal cord. In addition, the genetic ablation of FGF22 or its receptors limits the spontaneous formation of new synaptic connections between corticospinal collaterals and long propriospinal relay neurons and thereby impedes functional recovery following spinal cord injury (Jacobi *et al*, 2015).

Here, we now explore whether and how the targeted overexpression of FGF22 can be harnessed to therapeutically support synaptogenesis, circuit rewiring, and functional recovery after spinal cord injury. We use viral gene transfer in combination with conditional genetics and retrograde tracing to overexpress FGF22 into different

1 Institute of Clinical Neuroimmunology, University Hospital, LMU Munich, Munich, Germany
2 Biomedical Center Munich (BMC), Faculty of Medicine, LMU Munich, Planegg, Germany
3 Graduate School of Systemic Neurosciences, LMU Munich, Planegg, Germany
4 Elite Graduate Program M.Sc. Biomedical Neuroscience, TUM, Munich, Germany
5 Munich Cluster of Systems Neurology (SyNergy), Munich, Germany
*Corresponding author. Tel: +4989218071663; E-mail: florence.bareyre@med.uni-muenchen.de
†These authors contributed equally to this work
‡Present address: F.M. Kirby Neurobiology Center, Boston Children's Hospital, and Department of Neurology, Harvard Medical School, Boston, MA, USA

© 2023 The Authors. Published under the terms of the CC BY 4.0 license.

populations of spinal interneurons and show that FGF22 expression can guide improvements of circuit rewiring in the injured spinal cord. By initiating FGF22 gene transfer at different time points after injury and assessing the effects on functional recovery, we can further define a critical window following injury during which FGF22 therapy has to be initiated to affect motor recovery. Together our study reveals the potential of synaptogenic treatments to improve circuit rewiring and functional recovery and characterizes the spatial and temporal constraints of their application after spinal cord injury.

Results

Targeting FGF22 gene therapy to long propriospinal neurons enhances circuit rewiring

Following incomplete lesions of the spinal cord, the hindlimb corticospinal tract (hCST) extends collaterals into the cervical spinal cord where it forms intraspinal detour circuits by connecting to excitatory spinal relay interneurons, namely long propriospinal neurons (LPSN) that bypass the lesion site (Fig 1A). In order to specifically restrict FGF22 to LPSN, we developed a targeted gene therapy approach based on adeno-associated viruses (AAVs). Specifically we cloned the FGF22 open reading frame and EGFP separated by a p2A sequence into a AAV backbone in a Cre-dependent manner and under the control of a human synapsin promoter (Fig EV1A and B). *In vitro* infection of cortical neurons and quantification of the relative expression of FGF22 showed a more than 10^5 fold increase in mRNA expression of FGF22 demonstrating the potency and efficacy of our vector (Unpaired two-tailed *t*-test, $P = 0.0406$; Fig EV1C and D). We then injected a retrograde AAV expressing the Cre recombinase (retroAAV-Cre) into the lumbar spinal cord and the AAV expressing FGF22 under the control of a DIO sequence (rAAV-hSyn-DIO-FGF22-EGFP) into the cervical spinal cord, to obtain a specific FGF22 overexpression and labeling of LPSNs (Fig1B–D). We see about 13.04 ± 2.8 LPSNs transduced per section indicating that with this gene therapy strategy, we can direct FGF22 overexpression to a predefined subpopulation of cervical spinal interneurons. To analyze circuit formation following spinal cord injury, we first combined the labeling of LPSNs with anterograde labeling of the hCST using rAAV-mCherry injected 6 days prior to the injury. Since FGF22 is a presynaptic organizer, we first evaluated the density of excitatory boutons along the newly sprouting hCST collaterals. While FGF22 overexpression in LPSNs did not alter the density of boutons on these hCST collaterals (unpaired two-tails *t*-test, $P = 0.7634$), it led to a more than twofold increase in the number of vGlut1-expressing presynaptic boutons (unpaired two-tails *t*-test, $P = 0.0092$; Fig 1E and F) indicating an increased maturation of these excitatory boutons. As expected for an excitatory tract, no inhibitory presynaptic boutons were observed with the marker vGAT, and this did not change in response to FGF22 overexpression (unpaired two-tails *t*-test, $P = 0.1124$; Fig 1E and F). This is in line with previous reports that show that FGF22 organizes excitatory synapses during development (Terauchi *et al*, 2010) and demonstrate that overexpression of FGF22 can selectively increase the formation or maturation of excitatory synapses in the injured adult CNS. Next, we analyzed the formation of contacts between hCST collaterals and spinal LPSN. Here, we observed that FGF22 overexpression in LPSNs leads to a significant increase in the number of contacts formed by hCST collaterals onto these neurons (Mann–Whitney test, $P = 0.0476$; Fig 1G and H). In line with this observation and the results presented above, the total intensity of vGlut-positive inputs to LPSNs was increased as well confirming the enhanced formation of excitatory circuit inputs to these spinal relay neurons (Unpaired two-tailed *t*-test, $P = 0.0026$; Fig 1G and H).

Targeting FGF22 gene therapy to excitatory spinal neurons increases cervical CST connectivity and survival of lumbar motoneurons

As the formation of hCST-LPSN detour circuits is likely only one of multiple remodeling processes that go on in the injured spinal cord (Takeoka *et al*, 2014; May *et al*, 2017; Asboth *et al*, 2018; Engmann *et al*, 2020), we next explored whether targeting FGF22 to larger proportion of excitatory neurons in the spinal cord would lead to a broader enhancement of CST connections to the cervical spinal cord. For this purpose, we injected rAAV-hSyn-DIO-FGF22-EGFP into the cervical spinal cord of vGlut2-cre mice (Fig 2A). When we injected the rAAV expressing FGF22 (AAV-hSyn-DIO-FGF22-EGFP) into the cervical cord of vGlut2-Cre mice, we obtained a specific transduction of excitatory vGlut-positive neurons primarily located in the intermediate region of spinal cord (184.2 ± 35.1 neurons per section; Fig 2B). To analyze changes in the hCST targeting of these neurons following spinal cord injury, we combined the labeling of excitatory cervical interneurons with anterograde labeling of the CST using rAAV-mCherry as described above (Fig 2A). Consistent with our previous data, we observed that overexpression of FGF22 in excitatory interneurons significantly increases the probability of those neurons to be contacted by descending hCST collaterals, strengthening the fact that FGF22 expression is sufficient to promote the formation of excitatory contacts onto these neurons (Mann–Whitney test, $P = 0.0381$; Fig 2C). This is important as it is likely that not all these spinal interneurons are spontaneously involved in remodeling processes following SCI and broader FGF22 overexpression might recruit additional interneurons to rewiring CST circuits. We then reasoned that the transduction into cervical interneurons could also trigger effects remotely by strengthening input from LPSN onto other pools of neurons and focused in particular onto lumbar motorneurons. We observed that lumbar motoneuron survival was significantly increased after FGF22 overexpression in excitatory cervical spinal interneurons (Mann–Whitney test, $P = 0.0286$; Fig EV2A and B left). Interestingly, this effect was not apparent in case of overexpression of FGF22 in a much more restricted subset of cervical LPSNs (Unpaired two-tailed *t*-test, $P = 0.8551$; Fig EV2B right) indicating that a broad transduction of cervical neurons with FGF22 is required for this effect. The amount of synaptic puncta onto the remaining motoneurons was, however, not altered with either FGF22 overexpression approach (Fig EV2C–E).

Nonselective FGF22 gene therapy induces widespread circuit remodeling in the injured spinal cord

As broader expression of FGF22 thus resulted in broader support of spinal remodeling, we finally explored a nonselective FGF22 overexpression that could probably be most readily adapted to clinical

 © 2023 The Authors

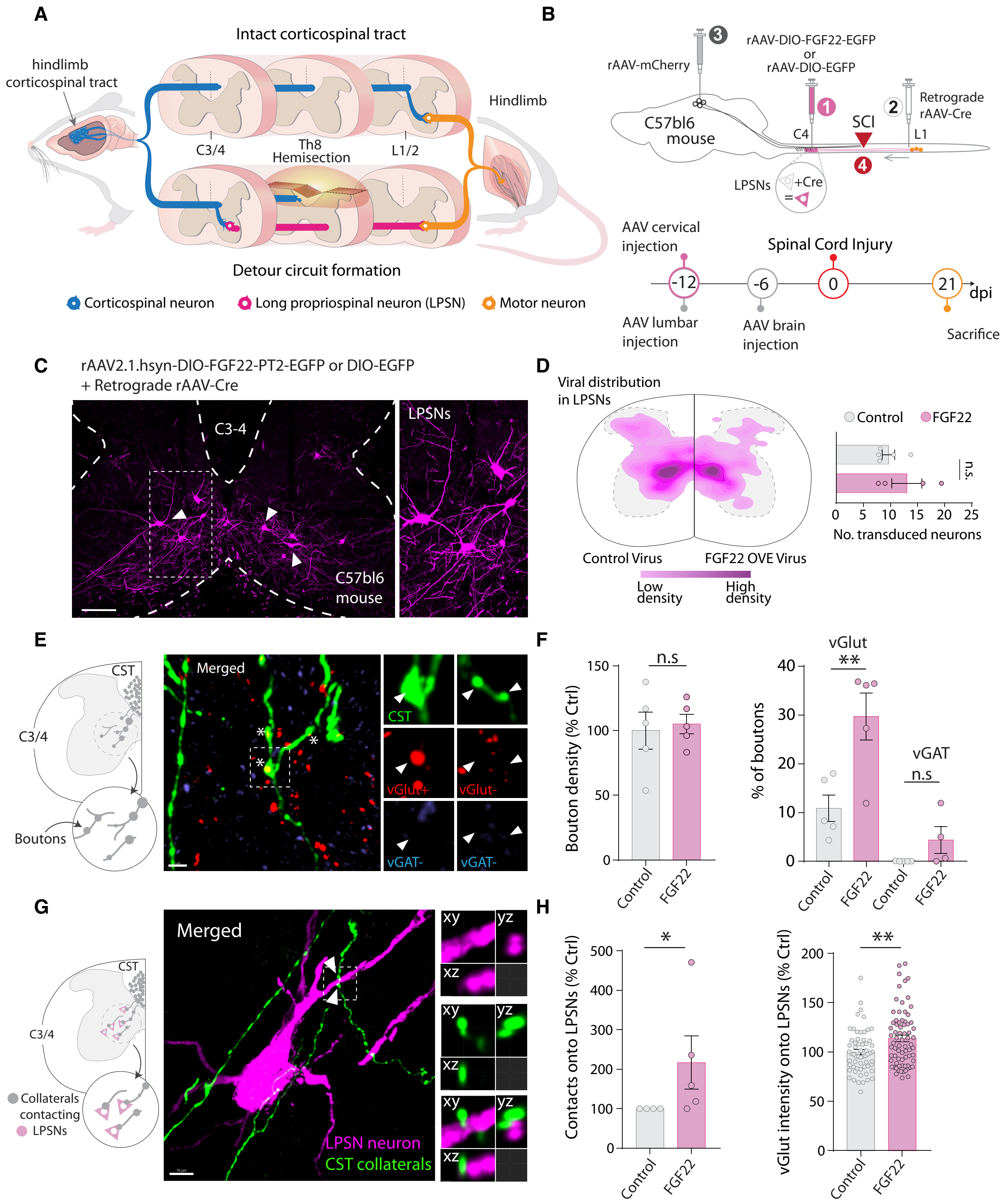
A
Intact corticospinal tract
hindlimb corticospinal tract
Hindlimb
C3/4
Th8 Hemisection
L1/2
Detour circuit formation
Corticospinal neuron
Long propriospinal neuron (LPSN)
Motor neuron
B
rAAV-mCherry
rAAV-DIO-FGF22-EGFP or rAAV-DIO-EGFP
Retrograde rAAV-Cre
C57bl6 mouse
C4
SCI
L1
LPSNs
+Cre
AAV cervical injection
Spinal Cord Injury
-12
-6
0
21
dpi
AAV lumbar injection
AAV brain injection
Sacrifice
C
rAAV2.1.hsyn-DIO-FGF22-PT2-EGFP or DIO-EGFP
+ Retrograde rAAV-Cre
C3-4
LPSNs
C57bl6 mouse
D
Viral distribution in LPSNs
Control
FGF22
n.s
No. transduced neurons
Control Virus
FGF22 OVE Virus
Low density
High density
E
CST
C3/4
Boutons
Merged
CST
vGlut+
vGlut-
vGAT-
vGAT-
F
Bouton density (% Ctrl)
n.s
vGlut
vGAT
% of boutons
Control
FGF22
G
CST
C3/4
Collaterals contacting LPSNs
Merged
xy
yz
xz
LPSN neuron
CST collaterals
H
Contacts onto LPSNs (% Ctrl)
vGlut intensity onto LPSNs (% Ctrl)
Control
FGF22

Figure 1.

Figure 1. Targeted FGF22 overexpression in long propriospinal neurons triggers excitatory synapse formation.

A Illustration of axonal plasticity of the hindlimb corticospinal tract (hCST; detour circuit formation) following spinal cord injury.
B Schematic illustration of the experiment to overexpress FGF22 into long propriospinal neurons (LPSN) and time line.
C Representative confocal image of long propriospinal neurons expressing FGF22 (magenta) in cervical spinal cord.
D Viral distribution of control and FGF22 overexpressing AAVs in long propriospinal neurons (LPSN) with number of transduced neurons (n = 4–5 mice per group).
E Schematic illustration of the hCST collateral into the cervical cord and confocal images of hCST collaterals (green), stained for vGlut (red) and vGAT (blue) 21 days following spinal cord injury. Insets represent 3D views generated in Imaris of the deconvolved confocal image.
F Quantification of bouton density and characterization of bouton between control and FGF22 overexpressing mice (n = 4–5 mice per group).
G Schematic illustration and representative confocal image of LPSN viral labeling (magenta) and CST collaterals (green). Insets represent 3D views generated in Imaris of the deconvolved confocal image.
H Quantification of hCST contacts onto LPSNs and characterization of vGlut intensity onto individual LPSNs (n = 4–5 mice per group).

Data information: ns: $P > 0.05$; $^*P < 0.05$ and $^{**}P < 0.01$. Unpaired t-test for panel (D) and Mann–Whitney test for panels (F, H). The data are presented as means ± SEM. Scale bar for (C) equals 300 µm; (E) and (G) equals 10 µm. Inset is magnified ~ 2 times in (C) and insets are magnified 4 times in (E) and (G). Arrowheads in (C) represent labeled LPSNs. Asterisk in (E) represent individual hCST boutons positive for vGlut staining. Arrowheads in G represent putative contacts between hCST and LPSN.

settings as it does not depend on the Cre-recombinase to restrict expression. To do so, we engineered a bicistronic construct including FGF22 and EGFP separated by an ires2 sequence into an AAV backbone under the control of a CMV promoter (Figs 3A and B and EV3A). We verified transfection- and infection-efficiency in cell culture and *in vivo* and can show successful targeting of neurons, including long propriospinal neurons (Fig EV3C) although we could also detect a substantial infection of glial cells, in particular

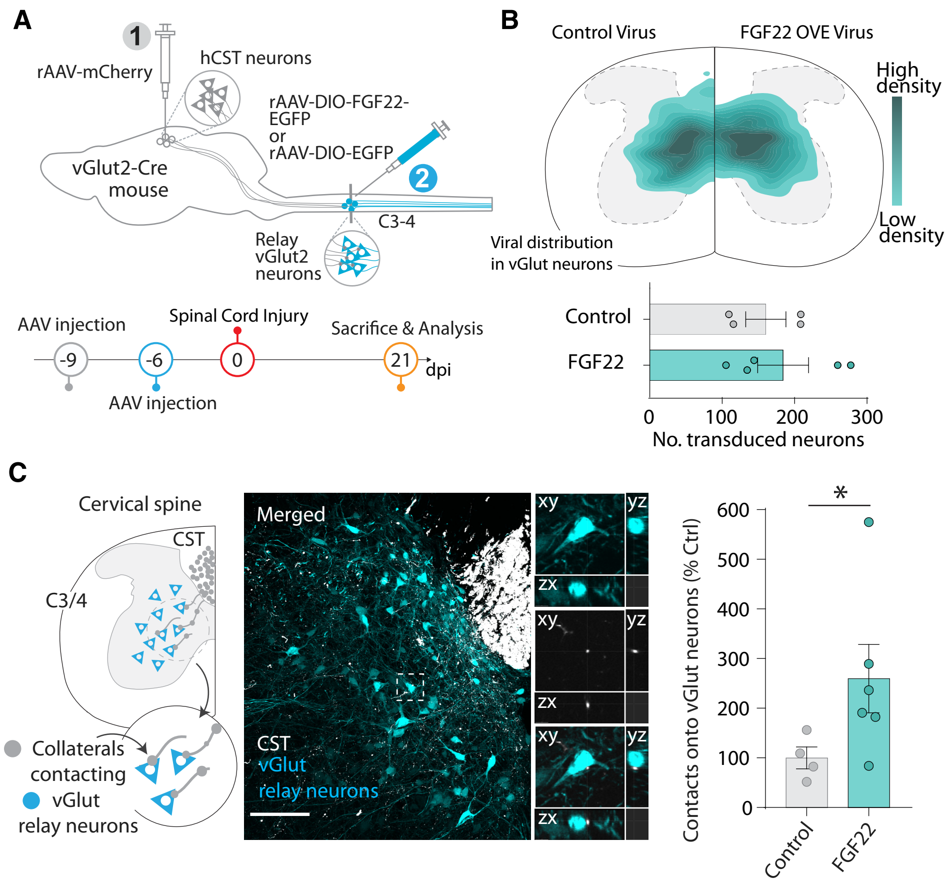

Figure 2. Targeted FGF22 overexpression in cervical excitatory neurons enhances hCST connectivity.

A Schematic illustration of the experiment to overexpress FGF22 into excitatory ($vGlut2^+$) cervical neurons and time line.
B Viral distribution of control and FGF22 overexpressing AAVs in excitatory interneurons with number of transduced neurons (n = 5–4 mice per group).
C Schematic illustration and representative confocal image for vGlut2-Cre neuronal labeling (cyan) and hCST collaterals (white). Insets represent 3D views generated in Imaris of the confocal image. Quantification of hCST contacts onto vGlut neurons (n = 4–6 mice per group).

Data information: ns: $P > 0.05$; $^*P < 0.05$. Unpaired t-test for panel (B) and Mann–Whitney test for panel (C). Scale bar for C equals 50 µm. Insets are magnified ~ 4 times in (C). The data are presented as means ± SEM.

 © 2023 The Authors

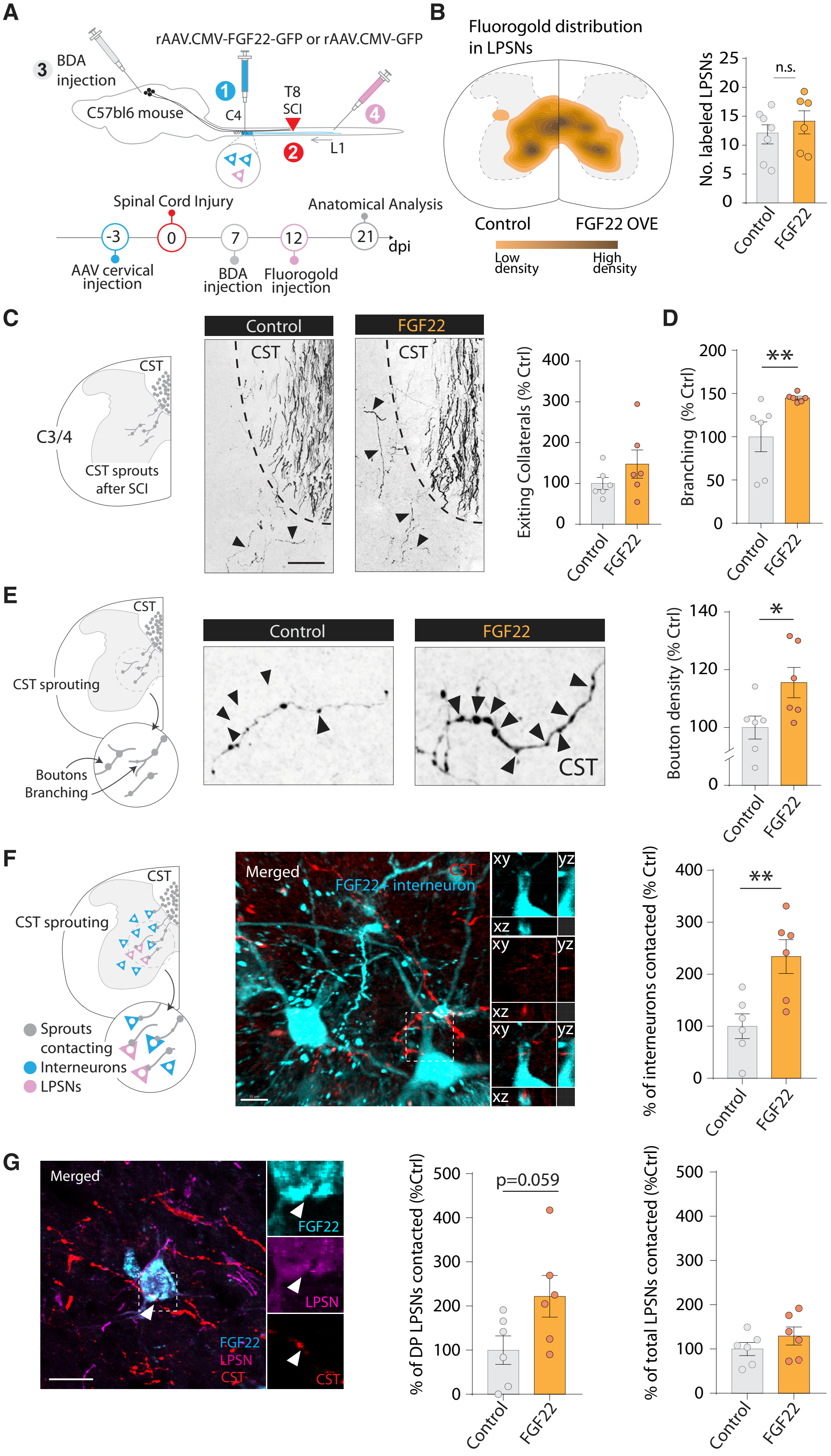
A
rAAV.CMV-FGF22-GFP or rAAV.CMV-GFP
3 BDA injection
1
C57bl6 mouse
C4
T8 SCI
4
2
L1
Spinal Cord Injury
Anatomical Analysis
-3
0
7
12
21
dpi
AAV cervical injection
BDA injection
Fluorogold injection
B
Fluorogold distribution in LPSNs
Control
FGF22 OVE
Low density
High density
No. labeled LPSNs
n.s.
Control
FGF22
C
C3/4
CST
CST sprouts after SCI
Control
FGF22
Exiting Collaterals (% Ctrl)
D
Branching (% Ctrl)
**
E
CST sprouting
Boutons
Branching
Bouton density (% Ctrl)
*
F
Sprouts contacting
Interneurons
LPSNs
Merged
FGF22+ interneuron
xy
yz
xz
% of interneurons contacted (% Ctrl)
**
G
Merged
FGF22
LPSN
% of DP LPSNs contacted (%Ctrl)
p=0.059
% of total LPSNs contacted (%Ctrl)

Figure 3.

◀ **Figure 3. Nonselective FGF22 gene therapy promotes widespread circuit rewiring.**

A Schematic illustration and time line of the experiment, in which we administered FGF22 before the spinal cord lesion.
B Schematic of the location of fluorogold-labeled LPSN in control and FGF22 overexpressing mice. Quantification of the number of fluorogold-labeled LPSN. Each dot represents individual animal (*n* = 6–7 animals per group).
C Schematic illustration of the hCST collateral exiting into the cervical cord and confocal images showing exiting hCST collaterals in control (left panel) and FGF22 treated (right panel) mice and quantification of the number of hCST collaterals that enter the cervical gray matter 3 weeks following spinal cord injury (*n* = 6 mice per group).
D Quantification of branching points on newly formed cervical hCST collaterals in control and FGF22 treated mice (*n* = 6 mice per group).
E Schematic illustration of the boutons on cervical hCST collateral and confocal images showing putative synaptic boutons (arrows) on newly formed cervical hCST collaterals at 3 weeks following spinal cord injury in a FGF22 (right) and a control (left) mice. Quantification of bouton density in control and FGF22 treated mice (*n* = 6 mice per group).
F Schematic illustration of the hCST contacts onto relay neurons in the cervical cord and confocal images showing contacts between CST collaterals (red) and cervical interneurons (cyan). Insets represent 3D views generated in Imaris of the confocal image. Quantification of the contacts between the hCST collaterals and cervical interneurons neurons (*n* = 6 per group).
G Confocal images showing contacts between CST collaterals (red) and long propriospinal neurons (magenta) transduced with FGF22 (cyan). Quantification of the contacts between the hCST collaterals and long propriospinal transduced with FGF22 or control virus (DP refers to double positive cells). Insets on the right are magnification of the boxed area on the left (*n* = 6 mice per group).

Data information: ns: $P > 0.05$; $^*P < 0.05$ and $^{**}P < 0.01$. Unpaired *t*-test for panels (E, F) and (G) and Mann–Whitney test for panel (D). The data are presented as means ± SEM. Scale bar equals 50 μm in panel (C); 10 μm in panel (F, G). Images in (E) are magnified five times from (C). Arrowheads in (C) represent exiting CST collaterals. Arrowheads in (D) represent bouton on hCST collaterals. Arrowhead in (G) represents putative contact between hCST collateral and FGF22 overexpressing LPSN.

astrocytes (see Materials and Methods). We first determined the potential of the new vectors to promote circuit remodeling and synaptogenesis by delivering rAAV-FGF22-EGFP prior to SCI (Fig 3A). We analyzed the effects on corticospinal connectivity following injury by quantifying the number of exiting collaterals, their boutons, and branching points into the cervical gray matter. While the number of exiting collaterals did not change as a results of global FGF22 overexpression (Unpaired two-tailed *t*-test, $P = 0.2357$; Fig 3C), the number of boutons and the complexity of the collaterals were markedly increased following treatment (Boutons: Unpaired two-tailed *t*-test, $P = 0.0393$; Branching: Mann–Whitney test, $P = 0.0087$; Fig 3D and E). We then analyzed the formation of contacts between CST collaterals and spinal interneurons. To do so, we made a distinction between the retrogradely labeled LPSN and other cervical interneurons. Interestingly, we demonstrate that overexpression of FGF22 induced a significant increase in contact formation between CST collaterals and spinal interneurons transduced with FGF22 (Unpaired two-tailed *t*-test, $P = 0.0076$; Fig 3F). To further analyze effects on CST-LPSN circuits, we retrogradely labeled LPSN in the cervical spinal cord and indeed found a selective increase in contacts onto FGF22 overexpressing LPSN (Unpaired two-tailed *t*-test, $P = 0.0594$; Fig 3G). Taken together, these results indicate that global overexpression of FGF22 can both increase the formation of CST-LPSN detour circuits and recruit additional spinal interneurons to the remodeling process together resulting in a marked enhancement of CST connectivity in the cervical spinal cord.

Defining a therapeutic window for FGF22 gene therapy

To test whether delivering FGF22 can also be therapeutically applied after SCI, we sought to assess recovery of motor function that could be achieved when global FGF22 gene therapy was delivered either immediately after the injury (acute treatment), 1 day after the injury (early treatment: 1 dpi), or 5-day postinjury (late treatment: 5 dpi). For all three application protocols, we evaluated functional recovery using the ladder rung test that evaluates fine paw placement (Liebetanz & Merkler, 2006; Loy *et al*, 2018; Aljovic *et al*, 2022; Fig 4A–E).

Figure 4. Postinjury therapeutic treatment with AAV-CMV-FGF22-ires-GFP triggers functional recovery in the ladder rung test. ▶

A Schematic illustration of the experimental design for therapeutic postinjury treatment with FGF22 following spinal cord injury.
B Illustration of regular (left) and irregular (right) ladder rung used to test functional recovery following spinal cord injury.
C Time line of the experiment with FGF22 injection right after the onset of the spinal cord lesion (top). Quantification of the functional recovery in the regular (middle) and irregular (bottom) ladder rung test in controls (gray) and FGF22 (orange) treated mice at baseline, at 3 days postinjury ("3 dpi"), 14 and 21 dpi after spinal cord injury (*n* = 9–10 mice per group).
D Time line of the experiment with FGF22 injection 24 h after the onset of the spinal cord lesion (top). Quantification of the functional recovery in the regular (middle) and irregular (bottom) ladder rung test in control (gray) and FGF22 (orange) treated mice at baseline, at 3 days postinjury ("3 dpi" or 2 days "2 dpi" for the late treatment), 14 and 21 dpi after spinal cord injury (*n* = 9–10 per group).
E Time line of the experiment with FGF22 injection 5 days after the onset of the spinal cord lesion (top). Quantification of the functional recovery in the regular (middle) and irregular (bottom) ladder rung test in control (gray) and FGF22 (orange) treated mice at baseline, at 3 days postinjury ("3 dpi"), 14 and 21 dpi after spinal cord injury (*n* = 9–10 per group).
F Representative confocal image of CST boutons (green) and synapsin staining (red) and quantification of the percentage of bouton synapsin positive (*n* = 6–7 mice per group).
G Representative confocal images of CST bouton (green) and bassoon staining (magenta) and quantification of the percentage of boutons that are bassoon positive (*n* = 5–6 mice per group).

Data information: Insets in (F) and (G) represent 3D views generated in Imaris of the confocal images. $^*P < 0.05$ and $^{**}P < 0.01$. ANOVA with the Šidak's *post hoc* test in (C–E) and *t*-test in (F, G). The data are presented as means ± SEM. Scale bars equals 10 μm in (F, G). Arrowhead in (F) represents hCST bouton expressing synapsin. Arrowhead in (G) represents hCST bouton expressing bassoon.

 © 2023 The Authors

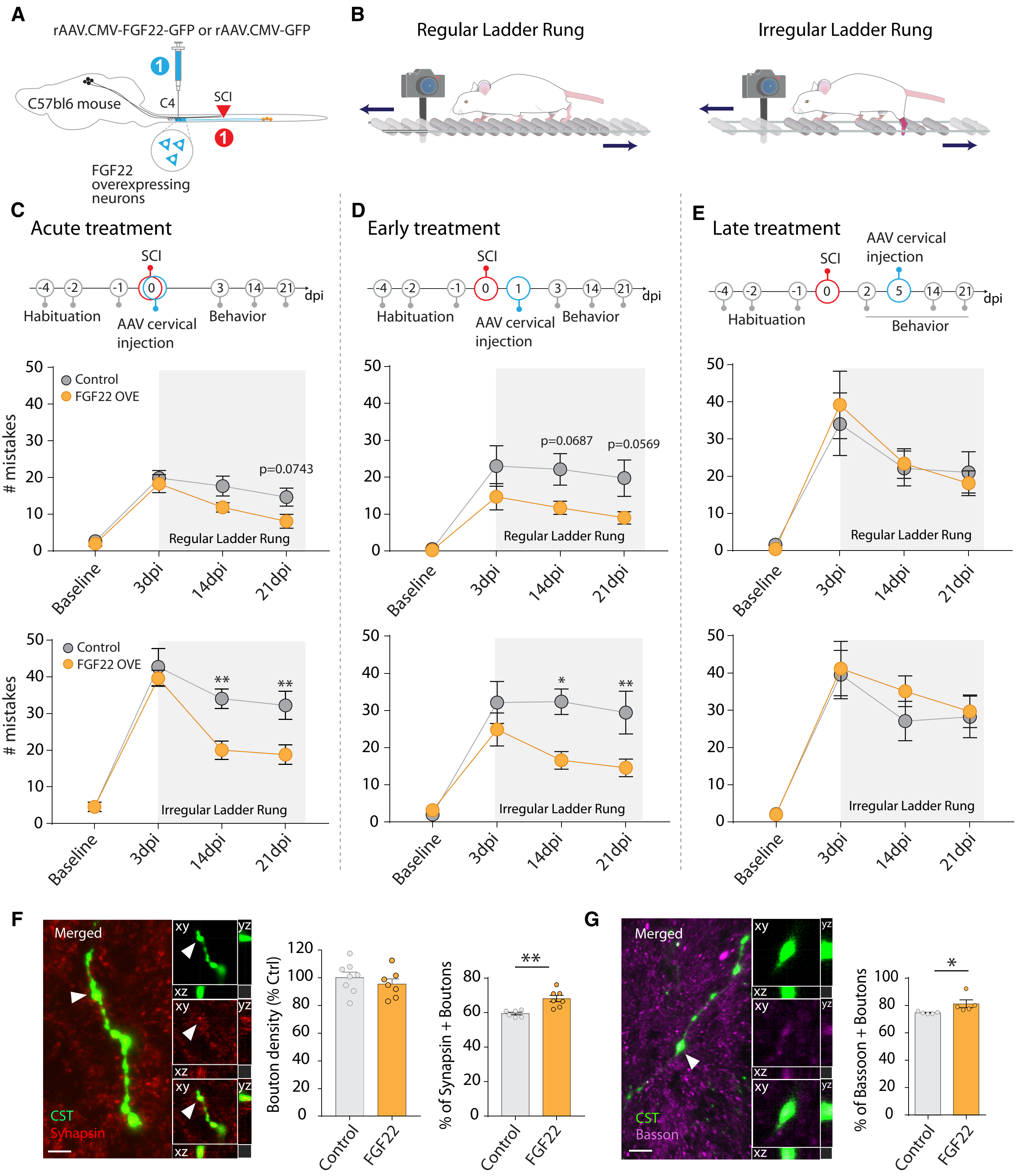

Figure 4.

We tested two ladder rung paradigms: In the first, the rungs are regularly spaced, to assess paw placements and general locomotion. In the second paradigm, the rungs are irregularly spaced to better assess the contribution of supraspinal input to sensory-motor integration during paw placement and locomotion. (Fig 4B). First, using chemogenetics and specific silencing of the hindlimb motor cortex,

we can show that recovery in the irregular ladder rung paradigm depends, to some extent, on the reorganization of the hindlimb motor cortex after injury (Fig EV4). Then, we used both recovery paradigms to study the effects of nonselective FGF 22 gene therapy initiated either immediately or 1 day after injury. In both cases, we observed an improved performance of the mice on the irregular ladder rung both at 14 and 21 dpi (Fig 4C and D) indicating that an enhanced corticospinal input to the lumbar motor circuits drives improved behavioral recovery (acute treatment: ANOVA followed by Sidak's test 14 dpi $P = 0.0061$; 21 dpi $P = 0.0093$; early treatment: ANOVA followed by Sidak's test 14 dpi $P = 0.0049$; 21 dpi $P = 0.0129$). In line with this interpretation, we did observe that early initiation of FGF22 gene therapy (at 1 day after injury) resulted in an enhanced maturation of presynaptic boutons along the CST collaterals as more boutons expressed the synaptic vesicle-associated protein synapsin and the active zone marker bassoon (Fig 4F and G; Synapsin: unpaired two-tailed t-test $P = 0.0028$; Bassoon: Mann–Whitney test, $P = 0.0159$). Notably, the effect of FGF22 gene therapy on behavioral recovery was lost when the treatment was started 5 days after the injury (Fig 4E), indicating that the additional time required for virus-mediated protein expression likely moves FGF22 delivery past the time period when new CST contacts are established (Lang *et al*, 2012). Taken together, our results reveal the presence of a critical therapeutic window, in at least the first 24 h after the spinal cord injury, during which synaptogenic gene therapy can improve the recovery of motor function.

Discussion

In this study, we used targeted gene therapy with rAAVs to overexpress FGF22, a presynaptic organizer, in different populations of spinal interneurons and determine the effect on circuit rewiring and functional recovery following spinal cord injury. We show that targeted gene therapy with FGF22 can improve circuit rewiring by inducing synaptogenesis and synapse maturation. We show that effects of FGF22 gene therapy are scalable as broader targeting strategies recruited additional spinal interneurons to the remodeling process. We further reveal that FGF 22 gene therapy, if initiated in the first 24 h after the injury, allows for a significant recovery of stepping and paw placements.

rAAV-based targeting strategies in the injured spinal cord

Here, we explored several approaches to selectively target FGF22 overexpression to different subpopulations of spinal neurons. We took advantage of adeno-associated viruses AAV2/1 and either drove the expression of FGF22 with a human synapsin or CMV promoter. Both constructs resulted in a reliable transduction of spinal interneurons around the site of injection in line with the neurotropism of the AAV2/1 (Wu *et al*, 2006; Srivastava, 2016; Büning & Srivastava, 2019). However, it should be noted that in the case of the CMV promoter, a sizable fraction of local astrocytes were transduced as well which can be avoided by neuron-specific targeting strategies. In the first part of the study, we therefore used Cre-dependent rAAVs to restrict expression to subsets of cervical interneurons, long propriospinal neurons, or excitatory interneurons, by either using a retrograde rAAV expressing the Cre recombinase or Vglut2-Cre mice. Moreover, systemic delivery strategies could for example be combined with cell-type-specific promoters such as mDLX for GABAergic neurons in the forebrain or CAMKII for cortical excitatory neurons to achieve comprehensive targeting of defined neuronal subpopulations (Haery *et al*, 2019). The continuous evolution of rAAV serotypes including the recent development of rAAV- PHP.eB further provides options for broad CNS targeting based on systemic virus injection (Chan *et al*, 2017; Dayton *et al*, 2018) and can allow the targeting of difficult cell type to infect namely microglia for example (Lin *et al*, 2022). Based on the availability of different serotypes, promoters as well as retrograde and conditional variants, rAAVs now provides a highly flexible tool set for experimental gene therapy in the central nervous system.

Synaptogenic gene therapy with FGF22 can both potentiate CST-LPSN detour circuits and recruit new interneurons to the spinal remodeling process

We have previously demonstrated that long propriospinal neurons—neurons that spontaneously form synaptic contacts with descending hindlimb CST collaterals in the cervical spinal cord following incomplete thoracic injuries—express FGF22 in adulthood (Jacobi *et al*, 2015). We show that targeted overexpression of this presynaptic organizer in subsets of spinal interneurons is sufficient to promote the formation of new synaptic contacts and thereby improve circuit remodeling and functional recovery in the injured spinal cord. Here, we specifically demonstrated the contribution of the hindlimb motor cortex to the recovery of distinct aspects of motor function using specific silencing with chemogenetics. Our results demonstrate that the hindlimb motor cortex and its rewiring contributes to part of the recovery of skilled paw placement following spinal cord injury. It is likely that remodeling processes of many supraspinal descending tracts and probably also of ascending sensory tracts are required to explain the recovery of sensory-motor function after injury.

It is now accepted that axonal plasticity at the lesion site and at distant spinal and supraspinal locations occur in the injured adult CNS, where they enable circuit rewiring and functional recovery (Weidner *et al*, 2001; Bareyre *et al*, 2004; Girgis *et al*, 2007; Courtine *et al*, 2008; Van Den Brand *et al*, 2012; Takeoka *et al*, 2014; Bradley *et al*, 2019; Takeoka & Arber, 2019). These newly formed circuits can be initiated spontaneously (Bareyre *et al*, 2004; Kerschensteiner *et al*, 2004) and can be enhanced using manipulations that foster axonal growth initiation (Yip *et al*, 2010; Bareyre *et al*, 2011; Lang *et al*, 2013; Jack *et al*, 2018), neutralize plasticity restrictions (García-Alías *et al*, 2009; Engmann *et al*, 2020), and stimulate activity-based rehabilitation (Van Den Brand *et al*, 2012; Shah *et al*, 2013; Asboth *et al*, 2018; Bonizzato *et al*, 2021). Our study demonstrates that a synaptogenic treatment approach delivered before or up to 1 day after the injury can foster circuit formation following spinal cord injury. FGF22 is a target-derived organizer of presynaptic differentiation that was shown to be critical for the establishment of new excitatory synapses during development and the organization of synapses in adulthood (Umemori *et al*, 2004; Terauchi *et al*, 2010; Pasaoglu & Schikorski, 2016; Li *et al*, 2020). As such, in adult mice, deletion of FGF22 was shown to trigger a decrease in hippocampal excitatory synapses and causes a

 © 2023 The Authors

depression-like phenotype (Williams *et al*, 2016). Furthermore, ablation of FGF22 in postsynaptic spinal interneurons or ablation of FGF22 receptors in the presynaptic CST collaterals indicated that FGF22 signaling important for the initial formation and maintenance of new circuits in the injured spinal cord (Jacobi *et al*, 2015). Here, we now show that this important endogenous regulator can also be harnessed for therapeutic purposes. In this context, it is interesting to note that not only contacts onto LPSN were increased but that a broad range of spinal interneurons could, once transduced with FGF22, attract additional CST synapses. This demonstrates the potential of FGF22 in promoting postinjury synapse formation but may also prompt us to be cautious regarding the development of potential miswiring involving, for example, unlesioned tracts such as the forelimb CST. Here, however, it is noteworthy that increased contacts were only observed in the neurons that overexpress FGF22 and not their nontransduced neighbors. This indicates that the diffusion of the secreted FGF22 is likely limited, maybe by binding to the extracellular matrix, resulting in the existence of tissue gradients as well-known, for example, for axon guidance molecules (Chédotal, 2019). The capability of FGF22 to attract contacts to defined neurons can be further harnessed to selectively target the rewiring process to defined neuronal circuits (as here demonstrated for CST-LPSN detour circuits). Notably, the local release of FGF22 will not only promote differentiation of synaptic boutons derived from hCST collaterals but also is very likely to also affect other supraspinal tracts who run in the vicinity of FGF22 overexpressing neurons and may enable them to contribute to postinjury circuit rewiring. In line with this assumption, the chemogenetic silencing of the hindlimb motor cortex we performed here indicates that part but not all the recovery following FGF22 treatment is mediated by the hindlimb motor cortex that encompasses the hCST.

Finally, it is important to note that FGF22 can have beneficial effects beyond supporting circuit rewiring. It has been for examples shown that FGF22 when delivered at the injury site immediately following spinal cord injury in mice can increase neuron survival, improve tissue density, and prevent ER stress-induced apoptosis (Zhu *et al*, 2020). This study thus demonstrates that local FGF22 delivery can support the survival of injured neurons at the site of injury, while our approach now demonstrates that FGF22 can also enhance the organization of synapses and the rewiring of healthy cells remote from the injury site. Synaptogenic therapies, of course, are in principle not restricted to FGF22 as a number of additional presynaptic organizers such as neuroligin or WNT7a have been identified (Hall *et al*, 2000; Scheiffele *et al*, 2000). In addition, most effective results might ultimately be obtained by a combination of different synaptogenic molecules as such molecules often act in concert as shown for cerebellar mossy fibers where several presynaptic organizers contribute to presynaptic differentiation (Hall *et al*, 2000; Scheiffele *et al*, 2000; Jacobi *et al*, 2014). Interestingly, we demonstrate that overexpression of FGF22 in all cervical excitatory relay neurons does not only increase presynaptic differentiation in cervical CST collaterals but also increase motoneuron survival in the lumbar cord. Although we could not detect an increase in contacts onto surviving motoneurons from the projecting cervical excitatory neurons, it is likely that more efficient contact formation between cervical spinal interneurons transduced with FGF22 increases the descending tonic input onto motoneurons, thereby enhancing their survival and preventing their degeneration (Han *et al*, 2019).

The first day(s) after injury represent a window of opportunity for FGF22 gene therapy

As our gene therapy approach is efficient to increase new circuit wiring following SCI, we then determined when such an approach has to be initiated to trigger functional recovery. We used the ladder rung test (Metz & Whishaw, 2009; Jacobi *et al*, 2015; Loy *et al*, 2018; Aljovic *et al*, 2022) which, among other parameters, provides a readout for successful CST rewiring since constant step-length adaptations requires supraspinal input (Liebetanz & Merkler, 2006). We demonstrate that when delivered at the time of injury or within 1 day later (1 dpi), gene therapy with FGF22 triggers a significant improvement in CST-dependent stepping abilities in the irregular ladder rung test. Performance in the regular ladder rung test showed a trend toward improvement that, however, did not reach significance. This is in line with previous observations demonstrating that rhythmic locomotion, as the one necessary for the regular ladder rung test, is known to be essentially under the control of spinal central pattern-generating networks, relying also on afferent cutaneous and proprioceptive feedback (Pearson, 1995; Gerasimenko *et al*, 2010; Klarner & Zehr, 2018). The findings that some improvement was observed here as well might indicate that rewiring of local circuits is enhanced by FGF22 therapy as well. Critically, however, all beneficial effects on ladder rung performance were lost when the administration of the rAAV gene therapy is delayed until 5-days postinjury.

This demonstrate the existence of a critical time window of endogenous axonal remodeling after injury, during which therapeutic intervention can influence the remodeling of new circuits and that is likely specific to the mode of action of the therapy. Since the action of FGF22 on the organization of the presynapse is relatively rapid at least *in vitro* (Terauchi *et al*, 2016), it is likely that its therapeutic action is primarily timed by the formation of new hCST collaterals into the cervical cord that takes about 1–2 weeks to establish (Lang *et al*, 2012). This time frame is likely to allow the overexpression of an adequate amount of FGF22 and its action to organize the presynaptic boutons (Terauchi *et al*, 2016) if viral gene transfer is induced shortly after the injury but not if treatment induction is delayed for longer times (in our case 5 days). This is in line with previous studies that demonstrate that the success of spinal cord therapies either pharmacological, cell-based, or rehabilitative that target circuit rewiring critically depends on their timing (Van Den Brand *et al*, 2012; Lang *et al*, 2013; Yu *et al*, 2013; Jacobi *et al*, 2015; Hilton *et al*, 2016; Kadoya *et al*, 2016; Han *et al*, 2019; Brommer *et al*, 2021). It would be interesting to explore whether the therapeutic window of synaptogenic genes therapies could be extended by combination with approaches that can re-open the critical window for circuit plasticity (Pizzorusso *et al*, 2002; Carulli *et al*, 2010; Morecraft *et al*, 2016; Duménieu *et al*, 2021). While such an extended therapeutic window would clearly further broaden the applicability of synaptogenic gene therapies, it is noteworthy that a postinjury window does appear to exist during which virus-based gene delivery can still modulate the endogenous remodeling process.

In summary, we demonstrate here that synaptogenic treatments like the viral delivery of FGF22 that promote the organization of the presynapse can foster the plasticity of healthy supraspinal axons in areas remote from the lesion and thereby contribute to recovery of function. Given the broad range of acute, subacute, and chronic neurological conditions in which synapse loss contributes to disease

© 2023 The Authors

pathology, such synaptogenic treatment concepts could prove to be of substantial biomedical relevance.

Materials and Methods

Mice

All animal procedures were performed according to the institutional guidelines and were approved by the Government of Upper Bavaria (animal protocol 55.2-1-54-2,532-135-15), and all the methods were performed in accordance with the relevant guidelines and regulations. Mice were maintained on a 12-h light/ 12-h dark cycle with food and water ad libitum. Adult female C57Bl/6j and adult female VGlut2-Cre (RRID:IMSR_JAX:016963) mice aged of 8–12 weeks at the start of the experiment (Vong *et al*, 2011) were used for this study. No sample size estimate was performed.

Plasmid design and virus production

pAAV-hSyn-DIO-FGF22-P2A-eGFP (rAAV-DIO-FGF22-P2A-eGFP) was created by inserting p2a-eGFP sequence from pLentiCas9-p2a-eGFP plasmid at the NcoI and BsrGI site of pAAV-hSyn-DIO-eGFP backbone. The coding sequence for FGF22 was amplified from pAAV-CMV-FGF22-ires2-hrGFP and inserted upstream of the P2a sequence. P2a-eGFP fragment was amplified from pLentiCas9-p2a-eGFP using the following primers: The forward primer (26-mer): TCTCGTCTGGATCCGGCGCAACAAAC and the reverse primer (38-mer): TATGGCGCGCCCTACTTGTACAGCTCGTCCATGCCGAG. The coding sequence for FGF22 was amplified from pAAV-CMV-FGF22-ires2-hrGFP plasmid by using the following primers: The forward primer (36-mer): GTTATGCTAGCGCCACCCCGGGCGGATCC-GAATTCG and the reverse primer (26-mer): CGGATCCAGACGA-GACCAAGACTGGC. Three fragments were assembled by using Gibson Assembly master mix (NEB E2611) according to the manufacturer protocol. Genomic titers were as following: rAAV-DIO-FGF22-P2A-eGFP, 4.4×10^{10} genome copies/ml; rAAV-DIO-eGFP, 1.1×10^{11} genome copies/ml.

pAAV-CMV-FGF22-Ires2-hrGFP (rAAV-FGF22-Ires2-hrGFP) was created by inserting an Ires sequence from pIres2-DsRed2 (BD Bioscience) at the HincII site of pAAV-CMV-MCS. The coding sequence for FGF22 was inserted upstream of the Ires sequence. Humanized Renilla reniformis green fluorescent protein (hrGFP) was excised and inserted downstream of the Ires sequence. The control pAAV-CMV-hrGFP (rAAV- hrGFP) used was created by exchanging the GFP sequence of the pAAV-CMV-GFP (a kind gift of Hildegard Büning; University of Cologne) with the same hrGFP sequence used above. Recombinant AAV chimeric virions containing a 1:1 ratio of AAV1 and AAV2 capsid proteins and the foreign gene were generated as previously described (Lang *et al*, 2013; Jacobi *et al*, 2015; Bradley *et al*, 2019). Genomic titers were as follows: rAAV-FGF22-Ires-hrGFP, 1.11×10^{12} genome copies/ml; rAAV-hrGFP, 1.1×10^{12} genome copies/ml.

Primary neuronal culture

We used postnatal day 1–3 vGlut2-Cre pups (The Jackson Laboratory) to dissect cortices. Enzymatic dissociation in 0.25% trypsin–EDTA supplemented with 100 U ml^{-1} DNAse, for 10 min at 37°C, was followed by mechanical dissociation. Cells were plated on poly-D-Lysine coated glass in 24-well plates. Cultures were kept in a 37°C, 5% CO_2 incubator in Neurobasal-A media supplemented with 0.5% penicillin/streptomycin, 0.5% B27 and 2 mM Glutamax. After 3 days *in vitro*, cells were infected with AAV2/1-hSyn-DIO-FGF22-EGFP and AAV2/1-hSyn-DIO-EGFP (~ 10,000 MOI). Media was changed every 3 days. Neurons were fixed at 14 days *in vitro*.

Surgical procedures

For all surgical procedures, mice were anesthetized with an i.p. injection of midazolam/medetomidin/fentanyl (Medetomidin 0.5 mg/kg, Orion Pharma; Midazolam 5.0 mg/kg, Ratiopharm; Fentanyl 0.05 mg/kg, B.Braun) on a heating pad (38°C). For pain management, meloxicam (Metacam®, Boeringer Ingelheim) was administered 6 h after antagonization and every 12 h for 72 h.

Thoracic dorsal hemisection

The skin over the vertebral column was incised, and the 8th thoracic vertebra was carefully exposed. A laminectomy was performed, followed by a dorsal hemisection of the spinal cord with fine iridectomy scissors as previously described (Loy *et al*, 2018; Bradley *et al*, 2019). This lesion bilaterally transects the main dorsal and minor dorsolateral corticospinal tract (CST), leaving the ventral white matter intact.

Stereotactic labeling of the hindlimb motor cortex

To label the hindlimb corticospinal tract (CST) fibers in the nonselective experiments, we pressure injected 1 µl of 10% biotinylated dextran amin (BDA, in 0.1% phosphate buffer [PB], 10,000 MW, Life Technologies) in the hindlimb motor cortex of each hemisphere. Stereotactic injections were performed 14 days prior to sacrifice with a finely pulled glass micropipette connected to a syringe at the following coordinates: −1.3 mm rostro-caudal, 1 mm lateral from bregma and 0.6 mm depth. The micropipette remained in place 3 min following the injection. In LPSN and vGlut experiments, labeling of the hindlimb motor cortex was caried out using rAAV8.hSyn.mCherry (Addgene, #114472-AAV8). 0.2 µl of the virus was injected using the same coordinates that were used for BDA labeling.

FGF22 overexpression in LPSNs

FGF22 was overexpressed in LPSN by pressure injection of 0.5 µl of rAAV-DIO-FGF22-P2A-eGFP in the third/fourth cervical segment of the spinal cord and injection of 0.4 µl of retrograde rAAV.hSyn.Cre (Addgene, #105553-AAVrg) in lumbar (L1/2) region of the spinal cord. The skin over the vertebral column at the 4th cervical vertebra was carefully exposed and laminectomy was performed. The dura was carefully removed, and rAAV-DIO-FGF22-P2A-eGFP was injected bilaterally into the spinal cord at the following coordinates: −0.4 mm from central vein and 0.9 mm depth. The same procedure was performed for the injection of the control virus (rAAV-DIO-eGFP). To express Cre recombinase in long propriospinal neurons, we first exposed the skin over the first lumbar spinal segment and performed laminectomy to expose the spinal cord. The dura was carefully removed and retrograde rAAV.hSyn.Cre (Addgene, #105553-AAVrg) was injected bilaterally into the spinal cord at the

 © 2023 The Authors

following coordinates: −0.3 mm from central vein and 0.8 mm depth. After each virus injection, the capillary micropipette was left in the spinal cord for 3 min and then carefully removed to prevent any backflow. In the hindlimb motor cortex silencing experiment, we overexpressed FGF22 in propriospinal neurons by systemic blood injection of rAAV.PHP.eB.hSyn.DIO.FGF22.EGFP that has the ability to cross the blood–brain barrier and infect all neurons (titer: 1×10^{12} vg/mouse). Cell specificity was achieved by retrograde AAV.hSyn.Cre (Addgene, #105553-AAVrg) injection in lumbar 1/2 region of spinal cord (Titer: 7×10^{12} vg/ml; 1:1 dilution; 0.2 µl per side). The virus was bilaterally injected into the spinal cord at the following coordinates: −0.4 mm from central vein and 0.9 mm depth. In this experiment, we used a PHP.eB systemic injection in order to increase the number of propriospinal neurons infected by targeting several segments of the cervical cord and obtain a behavioral readout.

FGF22-specific overexpression in vGlut2 neurons

FGF22 overexpression in vGlut2 neurons is performed by injection of 0.5 µl of rAAV-DIO-FGF22-P2A-eGFP in the 4th segment of the cervical spinal cord in vGlut2-Cre expressing mouse line (The Jackson Laboratory, #028863). In brief, the skin over the vertebral column at the 4th cervical vertebra was exposed following with laminectomy. After carefully removing dura, the rAAV-DIO-FGF22-P2A-eGFP was injected bilaterally into the spinal cord at the following coordinates: −0.4 mm from central vein and 0.9 mm depth. The capillary micropipette was left in the spinal cord for 3 min following the injection and then slowly removed to prevent any backflow. For the control group, the same procedure was performed using the rAAV-DIO-eGFP virus.

Labeling of long propriospinal neurons (LPSNs) in the nonselective overexpression experiment

Long propriospinal neurons were labeled by pressure injection of 0.5 µl of 2% Fluoro-Gold™ (Fluorochrome, LCC) in the first lumbar spinal segments of the spinal cord (thoracic vertebral level T12) 9 day prior to sacrifice. The skin was incised, and the space between the last thoracic and first lumbar vertebra was exposed. The dura was carefully opened, and Fluorogold was injected bilaterally into the spinal cord (coordinates from central vein ±0.6 mm, 0.8 mm depth). The capillary was left in place for 3 min after injection. rAAV-CMV-FGF22-hrGFP injection into the cervical cord were performed at various time points: Pretreatment: 3 days before T8 hemisection; Acute post-treatment: T8 hemisection and rAAV-CMV-FGF22-hrGFP injected at the same time; Therapeutic windows treatment: rAAV-CMV-FGF22-hrGFP injected 24 h after T8 hemisection; post-treatment: rAAV-CMV-FGF22-hrGFP injected 5 days after T8 hemisection. rAAV-CMV-FGF22-hrGFP transduced neurons, including long propriospinal neurons but also a sizable fraction of glial cells, in particular astrocytes (1/3 and 2/3 ratio respectively).

Silencing of hindlimb motor cortex

To silence the hindlimb motor cortex, we bilaterally injected rAAV-hSyn-hM4D(Gi)-DREADD-mCherry (Addgene: #50475-AAV8; Titer: 7×10^{12} vg/ml). Stereotactic injections were performed 2 weeks prior to behavioral testing with a finely pulled glass micropipette connected to a syringe at the following coordinates: −1.3 mm rostro-caudal, 1 mm lateral from bregma and 0.6 mm depth. Clozapine-N-Oxide (CNO) was delivered ip at 1 mg/kg.

Tissue processing and IHC

Tissue processing

Brains and the cervical enlargement C3-C5 of the spinal cords were dissected and postfixed overnight in PFA, then transferred to 30% sucrose/PBS solution overnight. 30 µm thick coronal sections of the cervical enlargement was cut using a cryostat, collected, and processed free floating. To visualize CST collaterals, the BDA signal was amplified by incubation with ABC Complex (Vector Laboratories) overnight at 4°C. After a 30-min tyramide amplification (Biotin-XX, TSA Kit #21, Life Technologies), sections were incubated overnight with Streptavidin-conjugated Alexa Fluor 647 or Streptavidin-conjugated Alexa Fluor 594 (1:500, Life technologies).

Immunohistochemistry

All primary antibodies were incubated at 4°C overnight. In cases where rAAV-mediated expression of GFP was restricted to 7 days, the GFP signal was amplified using an anti-GFP antibody (A11122; Life Technologies) followed by a Goat Anti-Rabbit-488 secondary antibody (Life Technologies; both 1:500). In order to characterizes contacts onto interneurons, we stained with Rabbit anti-vGlut1 (1:500; Synaptic Systems, #135303), Rabbit anti-vGlut1/2 (1:500; Synaptic Systems, #135503), Guinea pig anti-vGAT (1:1,000; Synaptic Systems, #131308), rabbit anti-Synapsin (1:500; Millipore), and mouse anti-Basson (1:200; ENZO) antibodies. In order to label moto neurons, we co-stained with rabbit anti-ChAT antibody (1:100; Abcam, #ab178850) and NeuroTrace435 (1:200; ThermoFisher, #N21479). Primary antibodies were diluted in 0.3% Triton 20 and PBS. All secondary antibodies were kept for 2 h at room temperature with gentle shaking. Secondary antibodies used are as follows: anti-rabbit AF647 (1:500; ThermoFisher, #A32795), anti-guinea pig Cy3 (1:500; Jackson ImmunoResearch, #706-165-148), anti-guinea pig AF633 (1:500; Sigma Aldrich, #SAB4600129).

Image acquisition and quantifications

All quantifications were performed by an observer blinded from treatment and injury status. Samples were coverslipped with Vectashield (Vector Laboratories). All imaging was performed at room temperature. Automated confocal scanning was performed with a FV10-ASW microscopy software on an upright Olympus FV1000 confocal microscope system and with a Leica SP8 system. We used standard filter sets, and acquisition settings were kept constant between control and treatment groups for each experiment. All analysis was performed blindly with respect to control and treatment groups.

Quantification of exiting BDA labeled CST collaterals into the gray matter

CST collaterals entering the cervical gray matter were imaged on a FV1000 Olympus confocal microscope (UPLSAPO 20×O/0.85) and counted on 10 consecutive z-stacks for each animals. The total length of CST collaterals and branching was calculated and normalized to the number of labeled CST axons. To correct for differences in interanimal tracing efficiency, we normalized the number of collaterals by the number of labeled fibers in the white matter dorsal column (from three sections) to express a ratio of exiting collaterals per main CST fibers.

© 2023 The Authors

Quantifications of boutons along CST collaterals

We used Leica SP8 system with HC PL APO 40×/1.30 Oil CS2 objective. Confocal images were first deconvolved using Huygens (SVI). Deconvolution used a theoretical point spread function based on the optical properties of the imaging system. For bouton density analysis, we used Imaris Software to quantify area of CST collaterals, followed by the spot detection tool in Imaris to quantify bouton number. Boutons were identified by their brightness, size, and rounded shape profile in relation to locally adjacent axonal structure. In particular, we defined boutons as rounded structures about three times brighter (pixel value) or at least about two times bigger (maximum Feret diameter) than their adjacent collateral and we trained experimenter to assess such structures. To determine bouton density, we divided the number of boutons by area of analyzed collaterals. To determine whether detected boutons are vGlut or vGAT positive, we used a defined threshold for staining intensity and spot detection tool in Imaris.

Contact analysis onto LPSN

For interneuron (LPSN and excitatory interneurons) imaging, we used an inverted Leica SP8 system with HC PL APO 40×/1.30 Oil CS2 objective. We used a 405 nm excitation laser with white light laser (470–670 nm) to scan tissue sections. To collect fluorescence emission, we used gated hybrid detectors and PMTs. In the LPSN experiment, GFP^+ cells were 3D reconstructed and rendered. Cell surface then was masked on the CST channel, and contacts were quantified using a spot detection tool. For vGlut and therapeutic treatment experiment, the analysis was manually performed using the Fiji Software. For the nonselective experiment, we used a FV1000 confocal microscope with a 40× objective to image and analyze the proportion of contacts between LSPN and CST collaterals in 10 consecutive sections of the cervical spinal cord (level C3–C5). The number of LPSN and of cervical interneurons contacted by CST collaterals as well as the total number of LPSN labeled and the total number of contacts were counted. The proportion of LPSN contacted by CST collaterals was then calculated as the ratio of all LPSN contacted by collaterals over the total number of LPSN. The number of total contacts was also calculated and normalized according to the number of labeled fibers in the main CST tract and labeled LPSN.

Motoneuron analysis

Prior to any image analysis, all images were randomly renamed using a custom-made script. This helped us minimize bias in the analysis. Cell counting plugin in Fiji was used to manually quantify the number of motoneurons. For vGlut quantification on motoneurons, we used Imaris. First $NeuroTrace^+$ and $ChAT^+$ cells were identified, and 3D surfaces rendered. The surface was masked on $vGlut^+$ channel. Finally, a spot detection tool in Imaris was used to quantify the number of $vGlut^+$ synapses.

Behavior analysis

Prior to behavioral testing, we habituated all animals on the regular ladder rung. In this task, mice are asked to cross a 1-m-long horizontal metal-rung runway with either regular gaps of 1 cm (regular walk) or varying gaps of 1–2 cm (irregular walk) between the rungs. One day before spinal cord injury, we recorded animals performing regular and irregular ladder rung tests. Mice had to perform three complete runs to be used in the analysis. A hindlimb error was defined as a complete miss or slip from the rung at the moment of the placement of the paw onto the rung. After spinal cord injury, mice were again tested at 14- and 21-day postinjury. In order to have unbiased analysis, all videos were renamed, and number of footfalls were quantified by two independent scientists. In the hindlimb motor cortex silencing experiment, upon recovery, mice were tested at 28-day postinjury followed by CNO (1 mg/kg) injection. 30-min post-CNO injection, animals were tasted again on the irregular ladder rung. We retested the mice after 24 h (Day 29) which represents at least a partial CNO washout (Rogers *et al*, 2021).

The paper explained

Problem

There is to date no therapy that can prevent the devastating consequences of spinal cord injury. While transected spinal axons are unable to spontaneously regenerate for long distances, the rewiring of neuronal circuits can enable functional recovery in the damaged central nervous system. Here, we investigate whether promoting such circuit rewiring by viral gene therapy with FGF22, a presynaptic organizer, can improve circuit plasticity and recovery of motor function following spinal cord injury.

Results

Here, we have targeted FGF22 gene therapy to either long propriospinal neurons, all excitatory interneurons, or spinal neurons in general to demonstrate differential effects on synapse formation and neuronal rewiring. We further demonstrate that such FGF22 gene therapy can also improve recovery of motor function after spinal cord injury. By initiating FGF22-targeted gene therapy at different postinjury time points, we also demonstrate the temporal constraints of its application and define a critical window for synaptogenic interventions.

Impact

Our study establishes that synaptogenic treatment strategies initiated within the first day after the insult can improve circuit rewiring and functional recovery following spinal cord injury. As synapse loss is a common feature of many neurological conditions, we propose that it is worthwhile to further explore the biomedical relevance of synaptogenic treatment strategies.

Statistical analysis

In all figures, results are presented as mean ± standard error of mean (SEM). Statistical analysis and graphical data illustration were performed using GraphPad Prism 7 for Windows (GraphPad Software). All data were tested for normality using the D'Agostino-Pearson omnibus normality test. For parametric data sets, we used unpaired *t*-test and for nonparametric data, we used Mann–Whitney test. In order to analyze behavioral outcomes, we were using repeated-measures ANOVA followed by Šidak's multiple comparisons test. Statistical significance levels are indicated as follows: $^*P < 0.05$; $^{**}P < 0.01$; $^{***}P < 0.001$.

© 2023 The Authors

Data availability

All data and codes generated in this study are available from the corresponding author upon reasonable request. This study includes no data deposited in external repositories.

Expanded View for this article is available online.

Acknowledgments

The authors would like to thank Bernadette Fiedler, Maria Goldberg, Michele Rosso, Maximilian Springer, Julie Fourneau, and Anja Schmalz for excellent technical assistance as well as Dana Matzek and Bianca Stahr for animal husbandry. We also thank the Core Bioimaging Facility of the Biomedical Center for their support and confocal imaging systems. We thank Laura Empl for excellent illustrations. Work in F.M.B.'s laboratory is supported by grants from: Deutsche Forschungsgemeinschaft (DFG, SFB870-ProjectID118803580, TRR274-ProjectID408885537). F.M.B. is also supported by the Munich Center for Systems Neurology (DFG, SyNergy; EXC2145/ID390857198). This project was specifically supported by the Wings for Life foundation. VVS is supported by a postdoctoral fellowship from the Humboldt foundation. Open access funding enabled and organized by Projekt DEAL.

Author contributions

Almir Aljović: Conceptualization; resources; data curation; software; formal analysis; supervision; validation; investigation; visualization; methodology; writing—original draft; project administration; writing—review and editing. **Anne Jacobi:** Conceptualization; resources; data curation; software; formal analysis; supervision; validation; investigation; methodology. **Maite Marcantoni:** Data curation; software; formal analysis; methodology. **Fritz Kagerer:** Data curation; formal analysis; methodology. **Kristina Loy:** Data curation; formal analysis; methodology. **Arek Kendirli:** Methodology. **Jonas Bräutigam:** Formal analysis. **Luca Fabbio:** Formal analysis. **Valérie Van Steenbergen:** Conceptualization; software; formal analysis; methodology. **Katarzyna Pleśniar:** Formal analysis. **Martin Kerschensteiner:** Conceptualization; writing—review and editing. **Florence M Bareyre:** Conceptualization; resources; data curation; formal analysis; supervision; funding acquisition; validation; investigation; methodology; writing—original draft; project administration; writing—review and editing.

Disclosure and competing interests statement

The authors declare that they have no conflict of interest.

References

Aljovic A, Zhao S, Chahin M, de la Rosa C, Van Steenbergen V, Kerschensteiner M, Bareyre FM (2022) A deep learning-based toolbox for automated limb motion analysis (ALMA) in murine models of neurological disorders. *Commun Biol* 51: 1 – 13

Asboth L, Friedli L, Beauparlant J, Martinez-Gonzalez C, Anil S, Rey E, Baud L, Pidpruzhnykova G, Anderson MA, Shkorbatova P *et al* (2018) Cortico-reticulo-spinal circuit reorganization enables functional recovery after severe spinal cord contusion. *Nat Neurosci* 21: 576 – 588

Bareyre FM, Kerschensteiner M, Raineteau O, Mettenleiter TC, Weinmann O, Schwab ME (2004) The injured spinal cord spontaneously forms a new intraspinal circuit in adult rats. *Nat Neurosci* 7: 269 – 277

Bareyre FM, Garzorz N, Lang C, Misgeld T, Büning H, Kerschensteiner M (2011) In vivo imaging reveals a phase-specific role of stat3 during central and peripheral nervous system axon regeneration. *Proc Natl Acad Sci USA* 108: 6282 – 6287

Bonizzato M, James ND, Pidpruzhnykova G, Pavlova N, Shkorbatova P, Baud L, Martinez-Gonzalez C, Squair JW, DiGiovanna J, Barraud Q *et al* (2021) Multi-pronged neuromodulation intervention engages the residual motor circuitry to facilitate walking in a rat model of spinal cord injury. *Nat Commun* 12: 1925

Bradley PM, Denecke CK, Aljovic A, Schmalz A, Kerschensteiner M, Bareyre FM (2019) Corticospinal circuit remodeling after central nervous system injury is dependent on neuronal activity. *J Exp Med* 216: 2503 – 2514

Brommer B, He M, Zhang Z, Yang Z, Page JC, Su J, Zhang Y, Zhu J, Gouy E, Tang J *et al* (2021) Improving hindlimb locomotor function by non-invasive AAV-mediated manipulations of propriospinal neurons in mice with complete spinal cord injury. *Nat Commun* 12: 781

Büning H, Srivastava A (2019) Capsid modifications for targeting and improving the efficacy of AAV vectors. *Mol Ther Methods Clin Dev* 12: 248 – 265

Carulli D, Pizzorusso T, Kwok JCF, Putignano E, Poli A, Forostyak S, Andrews MR, Deepa SS, Glant TT, Fawcett JW (2010) Animals lacking link protein have attenuated perineuronal nets and persistent plasticity. *Brain* 133: 2331 – 2347

Chan KY, Jang MJ, Yoo BB, Greenbaum A, Ravi N, Wu WL, Sánchez-Guardado L, Lois C, Mazmanian SK, Deverman BE *et al* (2017) Engineered AAVs for efficient noninvasive gene delivery to the central and peripheral nervous systems. *Nat Neurosci* 20: 1172 – 1179

Chédotal A (2019) Roles of axon guidance molecules in neuronal wiring in the developing spinal cord. *Nat Rev Neurosci* 20: 380 – 396

Courtine G, Song B, Roy RR, Zhong H, Herrmann JE, Ao Y, Qi J, Edgerton VR, Sofroniew MV (2008) Recovery of supraspinal control of stepping via indirect propriospinal relay connections after spinal cord injury. *Nat Med* 14: 69 – 74

Dabrowski A, Terauchi A, Strong C, Umemori H (2015) Distinct sets of FGF receptors sculpt excitatory and inhibitory synaptogenesis. *Development* 142: 1818 – 1830

Dayton RD, Grames MS, Klein RL (2018) More expansive gene transfer to the rat CNS: AAV PHP.EB vector dose–response and comparison to AAV PHP.B. *Gene Ther* 25: 392 – 400

Duménieu M, Marquèze-Pouey B, Russier M, Debanne D (2021) Mechanisms of plasticity in subcortical visual areas. *Cell* 10: 3162

Engmann AK, Bizzozzero F, Schneider MP, Pfyffer D, Imobersteg S, Schneider R, Hofer AS, Wieckhorst M, Schwab ME (2020) The Gigantocellular reticular nucleus plays a significant role in locomotor recovery after incomplete spinal cord injury. *J Neurosci* 40: 8292 – 8305

García-Alías G, Barkhuysen S, Buckle M, Fawcett JW (2009) Chondroitinase ABC treatment opens a window of opportunity for task-specific rehabilitation. *Nat Neurosci* 12: 1145 – 1151

Gerasimenko Y, Gorodnichev R, Machueva E, Pivovarova E, Semyenov D, Savochin A, Roy RR, Edgerton VR (2010) Novel and direct access to the human locomotor spinal circuitry. *J Neurosci* 30: 3700 – 3708

Girgis J, Merrett D, Kirkland S, Metz GAS, Verge V, Fouad K (2007) Reaching training in rats with spinal cord injury promotes plasticity and task specific recovery. *Brain* 130: 2993 – 3003

Haery L, Deverman BE, Matho KS, Cetin A, Woodard K, Cepko C, Guerin KI, Rego MA, Ersing I, Bachle SM *et al* (2019) Adeno-associated virus technologies and methods for targeted neuronal manipulation. *Front Neuroanat* 13: 93

© 2023 The Authors

Hall AC, Lucas FR, Salinas PC (2000) Axonal remodeling and synaptic differentiation in the cerebellum is regulated by WNT-7a signaling. *Cell* 100: 525 – 535

Han Q, Ordaz JD, Liu NK, Richardson Z, Wu W, Xia Y, Qu W, Wang Y, Dai H, Zhang YP *et al* (2019) Descending motor circuitry required for NT-3 mediated locomotor recovery after spinal cord injury in mice. *Nat Commun* 10: 5815

Hilton BJ, Anenberg E, Harrison TC, Boyd JD, Murphy TH, Tetzlaff W (2016) Re-establishment of cortical motor output maps and spontaneous functional recovery via spared dorsolaterally projecting corticospinal neurons after dorsal column spinal cord injury in adult mice. *J Neurosci* 36: 4080 – 4092

Jack AS, Hurd C, Forero J, Nataraj A, Fenrich K, Blesch A, Fouad K (2018) Cortical electrical stimulation in female rats with a cervical spinal cord injury to promote axonal outgrowth. *J Neurosci Res* 96: 852 – 862

Jacobi A, Schmalz A, Bareyre FM (2014) Abundant expression of guidance and synaptogenic molecules in the injured spinal cord. *PLoS One* 9: e88449

Jacobi A, Loy K, Schmalz AM, Hellsten M, Umemori H, Kerschensteiner M, Bareyre FM (2015) FGF 22 signaling regulates synapse formation during post-injury remodeling of the spinal cord. *EMBO J* 34: 1231 – 1243

Kadoya K, Lu P, Nguyen K, Lee-Kubli C, Kumamaru H, Yao L, Knackert J, Poplawski G, Dulin JN, Strobl H *et al* (2016) Spinal cord reconstitution with homologous neural grafts enables robust corticospinal regeneration. *Nat Med* 22: 479 – 487

Kerschensteiner M, Bareyre FM, Buddeberg BS, Merkler D, Stadelmann C, Brück W, Misgeld T, Schwab ME (2004) Remodeling of axonal connections contributes to recovery in an animal model of multiple sclerosis. *J Exp Med* 200: 1027 – 1038

Klarner T, Zehr EP (2018) Sherlock Holmes and the curious case of the human locomotor central pattern generator. *J Neurophysiol* 120: 53 – 77

Lang C, Guo X, Kerschensteiner M, Bareyre FM (2012) Single collateral reconstructions reveal distinct phases of corticospinal remodeling after spinal cord injury. *PLoS One* 7: e30461

Lang C, Bradley PM, Jacobi A, Kerschensteiner M, Bareyre FM (2013) STAT3 promotes corticospinal remodelling and functional recovery after spinal cord injury. *EMBO Rep* 14: 931 – 937

Li S, He J, Liu Y, Yang J (2020) FGF22 promotes generation of ribbon synapses through downregulating MEF2D. *Aging (Albany NY)* 12: 6456 – 6466

Liebetanz D, Merkler D (2006) Effects of commissural de-and remyelination on motor skill behaviour in the cuprizone mouse model of multiple sclerosis. *Exp Neurol* 202: 217 – 224

Lin R, Zhou Y, Yan T, Wang R, Li H, Wu Z, Zhang X, Zhou X, Zhao F, Zhang L *et al* (2022) Directed evolution of adeno-associated virus for efficient gene delivery to microglia. *Nat Methods* 19: 976 – 985

Loy K, Schmalz A, Hoche T, Jacobi A, Kreutzfeldt M, Merkler D, Bareyre FM (2018) Enhanced voluntary exercise improves functional recovery following spinal cord injury by impacting the local neuroglial injury response and supporting the rewiring of Supraspinal circuits. *J Neurotrauma* 35: 2904 – 2915

May Z, Fenrich KK, Dahlby J, Batty NJ, Torres-Espín A, Fouad K (2017) Following spinal cord injury transected Reticulospinal tract axons develop new collateral inputs to spinal interneurons in parallel with locomotor recovery. *Neural Plast* 2017: 1 – 15

Metz GA, Whishaw IQ (2009) The ladder rung walking task: a scoring system and its practical application. *J Vis Exp* 1204 https://doi.org/10.3791/1204

Morecraft RJ, Ge J, Stilwell-Morecraft KS, Mcneal DW, Hynes SM, Pizzimenti MA, Rotella DL, Darling WG (2016) Frontal and frontoparietal injury differentially affect the ipsilateral corticospinal projection from the nonlesioned hemisphere in monkey (*Macaca mulatta*). *J Comp Neurol* 524: 380 – 407

Pasaoglu T, Schikorski T (2016) Presynaptic size of associational/commissural CA3 synapses is controlled by fibroblast growth factor 22 in adult mice. *Hippocampus* 26: 151 – 160

Pearson KG (1995) Proprioceptive regulation of locomotion. *Curr Opin Neurobiol* 5: 786 – 791

Pizzorusso T, Medini P, Berardi N, Chierzi S, Fawcett JW, Maffei L (2002) Reactivation of ocular dominance plasticity in the adult visual cortex. *Science* 298: 1248 – 1251

Rogers S, Rozman PA, Valero M, Doyle WK, Buzsáki G (2021) Mechanisms and plasticity of chemogenically induced interneuronal suppression of principal cells. *Proc Natl Acad Sci USA* 118: e2014157118

Scheiffele P, Fan J, Choih J, Fetter R, Serafini T (2000) Neuroligin expressed in nonneuronal cells triggers presynaptic development in contacting axons. *Cell* 101: 657 – 669

Shah PK, Garcia-Alias G, Choe J, Gad P, Gerasimenko Y, Tillakaratne N, Zhong H, Roy RR, Edgerton VR (2013) Use of quadrupedal step training to re-engage spinal interneuronal networks and improve locomotor function after spinal cord injury. *Brain* 136: 3362 – 3377

Srivastava A (2016) In vivo tissue-tropism of adeno-associated viral vectors. *Curr Opin Virol* 21: 75 – 80

Takeoka A, Arber S (2019) Functional local proprioceptive feedback circuits initiate and maintain locomotor recovery after spinal cord injury. *Cell Rep* 27: 71 – 85.e3

Takeoka A, Vollenweider I, Courtine G, Arber S (2014) Muscle spindle feedback directs locomotor recovery and circuit reorganization after spinal cord injury. *Cell* 159: 1626 – 1639

Terauchi A, Johnson-Venkatesh EM, Toth AB, Javed D, Sutton MA, Umemori H (2010) Distinct FGFs promote differentiation of excitatory and inhibitory synapses. *Nature* 465: 783 – 787

Terauchi A, Johnson-Venkatesh EM, Bullock B, Lehtinen MK, Umemori H (2016) Retrograde fibroblast growth factor 22 (FGF22) signaling regulates insulin-like growth factor 2 (IGF2) expression for activity-dependent synapse stabilization in the mammalian brain. *Elife* 5: e12151

Umemori H, Linhoff MW, Ornitz DM, Sanes JR (2004) FGF22 and its close relatives are presynaptic organizing molecules in the mammalian brain. *Cell* 118: 257 – 270

Van Den Brand R, Heutschi J, Barraud Q, DiGiovanna J, Bartholdi K, Huerlimann M, Friedli L, Vollenweider I, Moraud EM, Duis S *et al* (2012) Restoring voluntary control of locomotion after paralyzing spinal cord injury. *Science* 336: 1182 – 1185

Vong L, Ye C, Yang Z, Choi B, Chua S, Lowell BB (2011) Leptin action on GABAergic neurons prevents obesity and reduces inhibitory tone to POMC neurons. *Neuron* 71: 142 – 154

Weidner N, Ner A, Salimi N, Tuszynski MH (2001) Spontaneous corticospinal axonal plasticity and functional recovery after adult central nervous system injury. *Proc Natl Acad Sci USA* 98: 3513 – 3518

Williams AJ, Yee P, Smith MC, Murphy GG, Umemori H (2016) Deletion of fibroblast growth factor 22 (FGF22) causes a depression-like phenotype in adult mice. *Behav Brain Res* 307: 11 – 17

Wu Z, Asokan A, Samulski RJ (2006) Adeno-associated virus serotypes: vector toolkit for human gene therapy. *Mol Ther* 14: 316 – 327

Yip PK, Wong LF, Sears TA, Yáñez-Muñoz RJ, McMahon SB (2010) Cortical overexpression of neuronal calcium sensor-1 induces functional plasticity in spinal cord following unilateral pyramidal tract injury in rat. *PLoS Biol* 8: e1000399

Yu F, Wang Z, Tanaka M, Chiu CT, Leeds P, Zhang Y, Chuang DM (2013) Posttrauma cotreatment with lithium and valproate: reduction of lesion volume, attenuation of blood-brain barrier disruption, and improvement

 © 2023 The Authors

in motor coordination in mice with traumatic brain injury. *J Neurosurg* 119: 766 – 773

Zhu S, Chen M, Chen M, Ye J, Ying Y, Wu Q, Dou H, Bai L, Mao F, Ni W *et al* (2020) Fibroblast growth factor 22 inhibits ER stress-induced apoptosis and improves recovery of spinal cord injury. *Front Pharmacol* 11: 18

License: This is an open access article under the terms of the Creative Commons Attribution License, which permits use, distribution and reproduction in any medium, provided the original work is properly cited.

Expanded View Figures

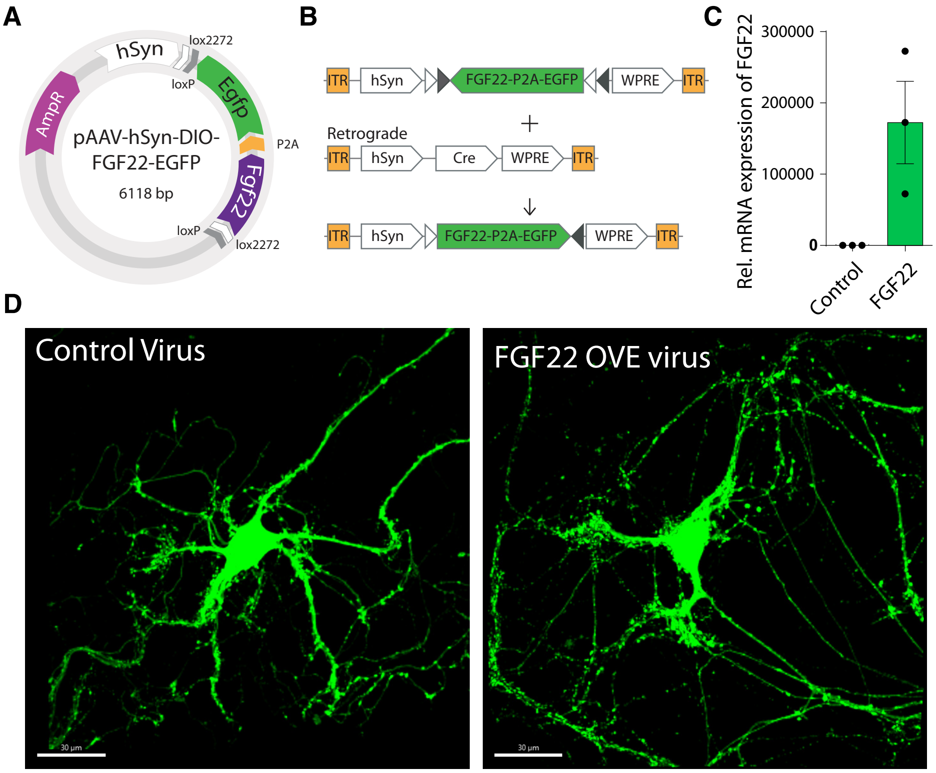

Figure EV1. Testing viral constructs in primary cortical neurons.

A, B Illustration of plasmid construct and double inverted cre-lox system for conditional FGF22 overexpression.

C qPCR quantification of Fgf22 overexpression in cultured HEK cells ($n = 3$ replicates per condition). Data are expressed as mean ± SEM.

D Representative confocal images of *in vitro* cortical neurons from vGlut2-cre mice (green) infected with (left) rAAV-hSyn-DIO-EGFP and (right) with rAAV-hSyn-DIO-FGF22-EGDP. Scale bar equals 20 μm.

 © 2023 The Authors

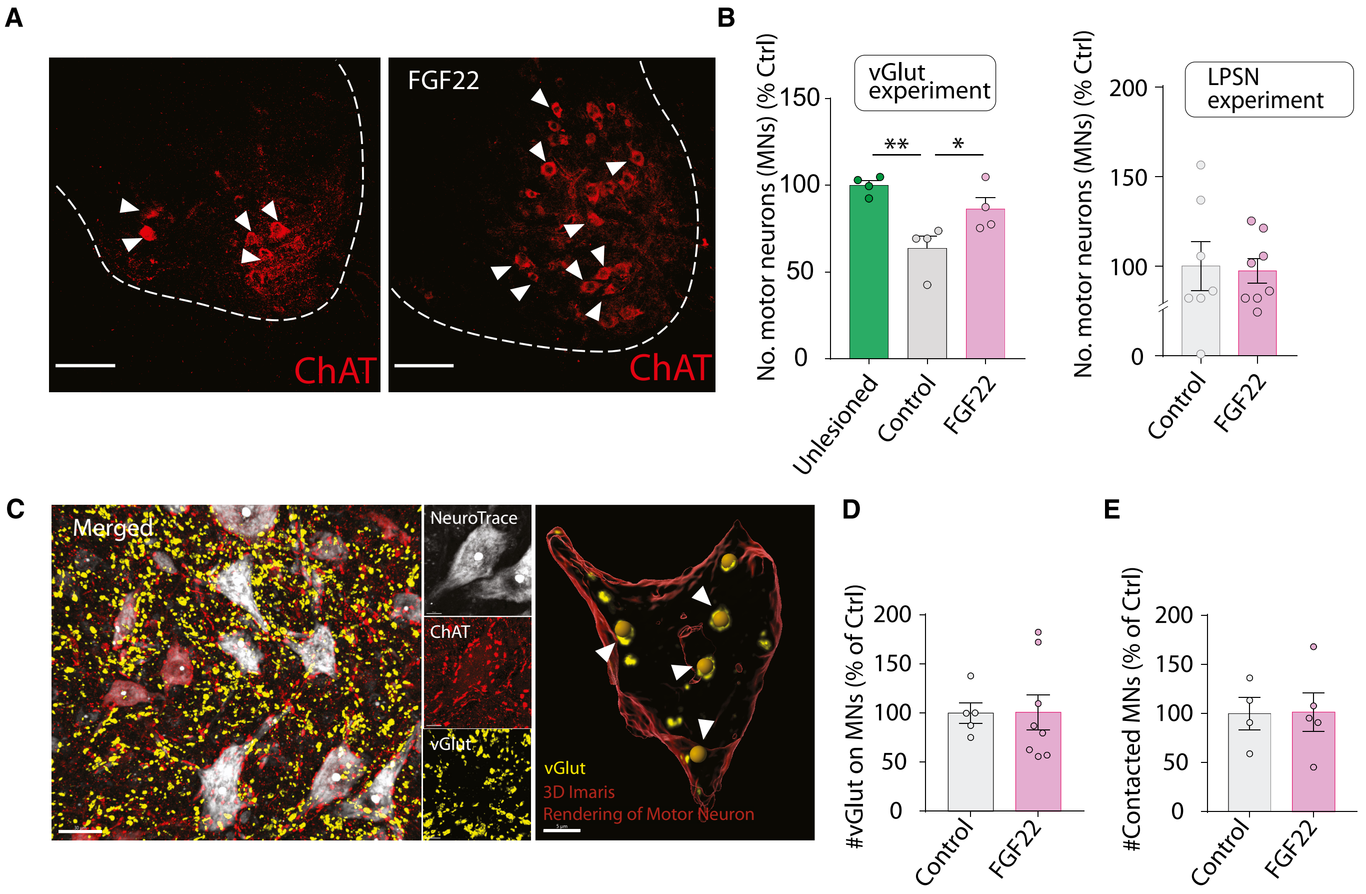

Figure EV2. FGF22 triggers motoneuron survival when delivered to vGlut-cre mice.

- A Representative confocal images of ChAT-based motoneuron immunostaining (red).
- B Quantification of motoneuron survival following FGF22 overexpression in excitatory neurons (left) and LPSN (right) (4–8 mice per group; green: unlesioned mice; gray: lesioned mice treated with control virus and magenta: lesioned mice treated with FGF22 virus).
- C (Left) Representative confocal images of motoneurons (red: ChAT staining and white: Neurotrace 435) and vGlut staining (yellow). (Right) 3D surface rendered in Imaris Software, showing quantification of vGlut puncta on a motor neuron.
- D Quantification of vGlut spots onto motoneurons, normalized to neuron surface area in LPSN experiment. Results are represented as percentage of control ($n = 5$–8 animals per group).
- E Quantification of vGlut projections onto motoneurons. Results are represented as percentage of control ($n = 4$–5 per group).

Data information: $^{*}P < 0.05$ and $^{**}P < 0.01$. Unpaired t-test for panel (B) (unlesioned vs. control) and Mann–Whitney test for panel (B) (control vs. FGF22). Unpaired t-test for panel (B) (LPSN experiment) and panels (D) and (E). Scale bar for (A) equals 50 μm and for (C) left 30 μm and for (C) right 5 μm. Arrowheads in (A) represent ChAT^{+} motoneurons. Arrowheads in (C) represent vGlut contact onto motoneuron. Data are expressed as mean ± SEM.

© 2023 The Authors

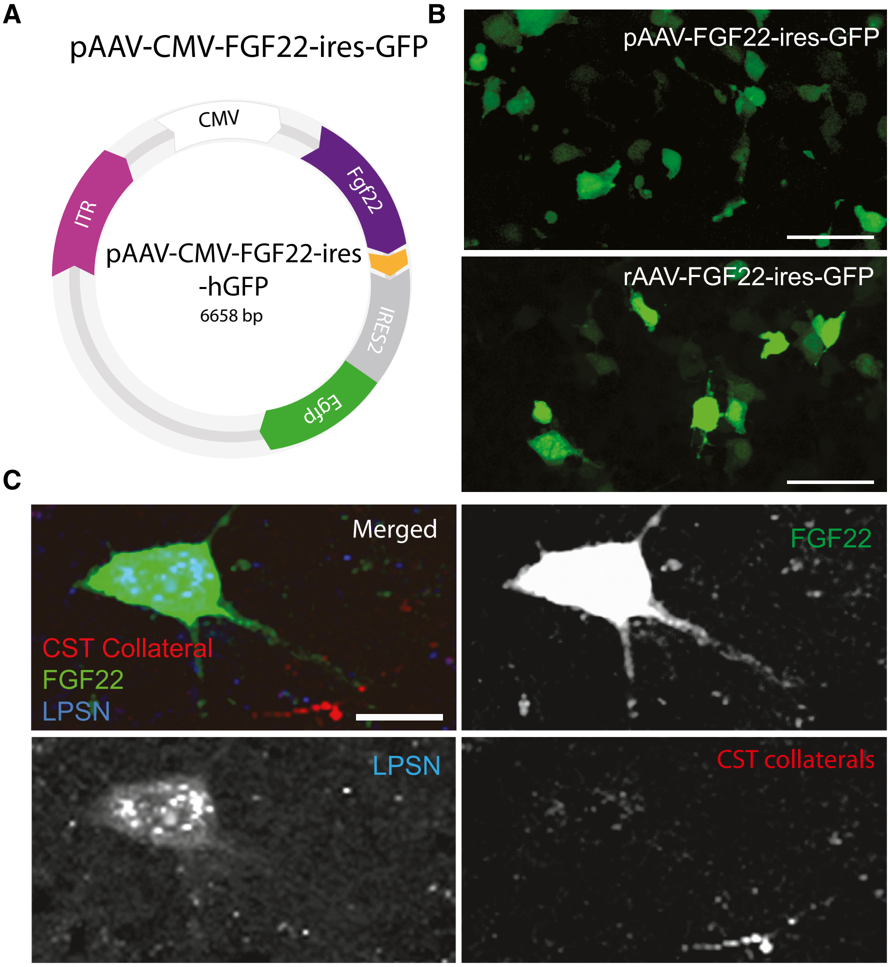

Figure EV3. Design and validation of plasmid used for testing therapeutic potentials.

A Illustration of plasmid construct used for therapeutic treatment.

B Representative images of plasmids tested in HEK cells.

C Representative confocal images of long propriospinal neuron retrogradely labeled with FluoroGold (blue), FGF22 overexpression (green) and CST collateral (white) contacting FGF22 overexpressing neuron.

Data information: Scale bar in (B) equals ~ 30 µm and (C) equals 5 µm.

 © 2023 The Authors

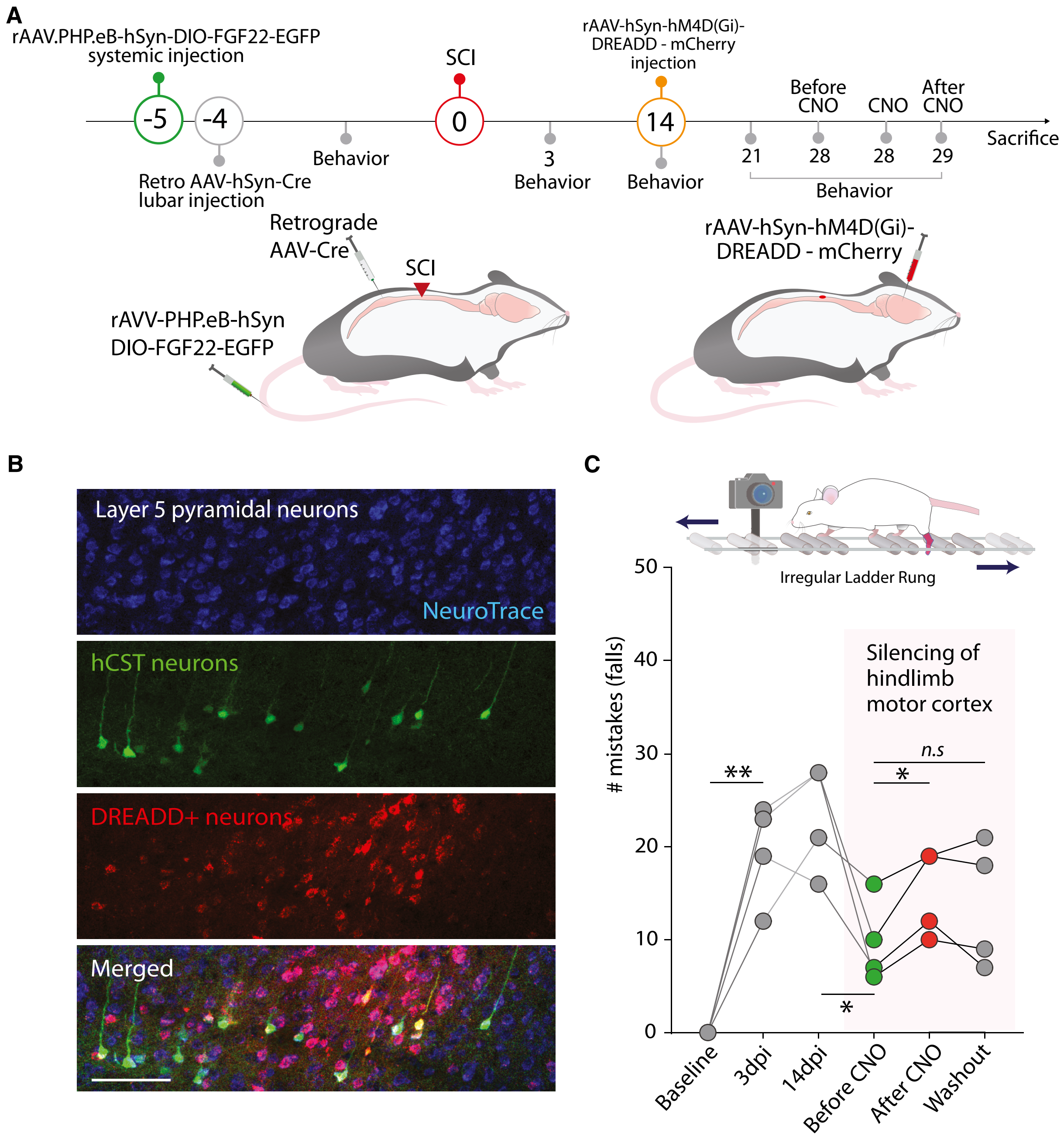

Figure EV4. Chemogenetic silencing of the hindlimb motor cortex demonstrates its contribution to functional recovery following spinal cord injury.

A Time line of the experiment in which FGF22 is overexpressed in long propriospinal neurons and silencing DREADDs are delivered to the hindlimb motor cortex.

B Confocal images of the DREADDs expressing neurons retrogradely labeled with AAV-Cre (Neurotrace: blue; Retrogradely labeled neurons: green; DREADD+ neurons: red). Scale bar equals 100 µm.

C Longitudinal quantifications of mistakes in the irregular ladder rung at baseline, 3-day postinjury (dpi), 14 dpi, 28 dpi before CNO administration (green dots), 28 dpi 30 min after CNO administration (red dots) and 29 dpi (CNO washout; gray dots) and scheme of the ladder rung task.

Data information. ns: $P > 0.05$; $^{*}P < 0.05$ and $^{**}P < 0.01$. For general group difference one-way repeated-measures ANOVA was used. In order to assess recovery of individual mice paired t-test was used (panel C).

© 2023 The Authors
