# Supplementary material for: Synaptogenic gene therapy with FGF22 improves circuit plasticity and functional recovery following spinal cord injury
Source: EMBO Mol Med. 2023 Jan 5;15(2):e16111. doi: 10.15252/emmm.202216111 (PMC9906383; doi:10.15252/emmm.202216111)
Supplement: Supplementary file 3 [file EMMM-15-e16111-s002.pdf]

EMBO Molecular Medicine

Synaptogenic gene therapy with FGF22 improves circuit plasticity and functional recovery following spinal cord injury

Almir Aljović, Anne Jacobi, Maite Marcantoni, Fritz Kagerer, Kristina Loy, Arek Kendirli, Jonas Bräutigam, Luca Fabbio, Valérie Van Steenbergen, Katarzyna Pleśniar, Martin Kerschensteiner, and Florence Bareyre
DOI: 10.15252/emmm.202216111

Corresponding author(s): Florence Bareyre (Florence.Bareyre@med.uni-muenchen.de)

Review Timeline:

Submission Date:	1st Apr 22
Editorial Decision:	27th Apr 22
Revision Received:	10th Nov 22
Editorial Decision:	1st Dec 22
Revision Received:	14th Dec 22
Accepted:	15th Dec 22

Editor: Jingyi Hou

Transaction Report:

(Note: With the exception of the correction of typographical or spelling errors that could be a source of ambiguity, letters and reports are not edited. Depending on transfer agreements, referee reports obtained elsewhere may or may not be included in this compilation. Referee reports are anonymous unless the Referee chooses to sign their reports.)

1st Editorial Decision 27th Apr 2022

Thank you again for submitting your work to EMBO Molecular Medicine. We have now heard back from the three referees who agreed to evaluate your manuscript. As you will see from the reports below, the referees acknowledge the potential interest of the study. Still, they raise a series of concerns, which we would ask you to address in a revision of the manuscript.

Without reiterating all the points raised in the reviews below, some of the more substantial issues are the following:

- Both Referees #1 and #3 mention that the direct evidence demonstrating that the observed motor functional recovery is mediated by FGF22-induced spinal cord circuit rewiring remains insufficient. We would encourage you to provide at least some levels of additional experimental evidence in this regard to improve the overall advance of the study, and overstatement should be avoided, as suggested by Referee #1.

- In line with Referee #2's comment regarding Zhu et al., attention should be given to placing the findings in the context of existing literature and highlighting the novelty of the current study.

Other issues raised by the referees need to be addressed as well. During our pre-decision cross-commenting process (in which the referees are given a chance to make additional comments, including on each other's reports), they made further comments, which I have included below after the referees' reports.

***** Reviewer's comments *****

Referee #1 (Remarks for Author):

Aljović and colleagues report on a molecular approach to enhance synapse remodeling after spinal cord injury. This work builds on their previous studies showing that corticospinal axons transected in the thoracic spinal cord sprout collaterals within the cervical cord and that the maturation of synaptic contacts made by these collaterals is dependent on fibroblast growth factor 22 (FGF22) signaling. Here they demonstrate that virally mediated FGF22 expression in the cervical spinal cord enhances maturation of novel contacts on long propriospinal neurons after thoracic injury. These propriospinal neurons have been established as critical drivers of hindlimb recovery after thoracic injury in rodent models. Furthermore, the authors find that viral transduction within the first day after injury can support recovery of hindlimb stepping on the irregular ladder test. The manuscript is well written, and the viral transduction and tracing methodology are well thought out. I have some concerns over interpretation of the findings and some missing methodological description, particularly as pertaining to poly-synaptic motor neuron loss. Overall, the study builds nicely on prior research output and provides a unique approach to enhancing formation of novel relay circuits after spinal cord injury.

The manuscript is largely focused on the role of the CST and corticospinal remodeling is implicated in the observed behavioral recovery. Surprisingly, no manipulations (optogenetic, chemogenetic, transection, conditional deletion of FGFR1R2, etc.) were made to test the CST contribution to recovery. The role for CST synaptic remodeling in functional recovery that is discussed in the results section is more speculative and would best be moved to the discussion.

The proposed loss of lumbar motor neurons following thoracic injury is surprising. The degeneration described in the Han paper referenced (#43), and the study that it refers to, show no loss of lumbar motor neurons, but a dendritic atrophy and synaptic loss. What were the specific levels at which lumbar motor neurons were counted? Were stereological counts or corrections made to account for over or under sampling? What are the total projected numbers of motor neurons at these lumbar levels and do you have intact numbers of motor neurons in the same area to demonstrate the extent of expected cell loss?

The authors propose that the similar recovery levels on regular and irregular ladder crossing with early AAV2/1-CMV-FGF22-IRES-GFP transduction may be due to distinct mechanisms on local versus descending circuit remodeling. It is more likely that these effects are related, with a reasonable interpretation being that supraspinal input enhances sensory-motor integration on

the irregular ladder behavior. This behavior also raises the question of how does FGF22 over-expression affect presynaptic input from other populations of neurons (supraspinal, local, primary afferent)?

The study on post-injury delivery of AAV2/1-CMV-FGF22-IRES-GFP nicely illustrates the time window for therapeutic intervention aimed at enhancing endogenous plasticity and circuit remodeling. How long after transduction are levels of FGF22 sufficient to induce synaptic maturation detected?

What do numbers 1-4 mean in Figure 1B? They are not the order of procedures, as they are in 2A and 3A.

Is the arrowhead in 1G meant to indicate a point of contact? If so, it doesn't appear to indicate a bouton-like pre-synaptic terminal.

As the quantification in 1F indicates that only approximately 5-37% of boutons show vGlut1 staining, would you include a representative image of CST boutons without vGlut expression?

Why do the normalized CST-LPSN contacts (1H) in controls show no variability, unlike other normalized control values?

Are the number of samples in vGlut intensity plot in 1H indicating individual puncta? The context and interpretation of this data is not clear.

The truncated y-axis in Fig 3E prevents the interpretation of the control data.

The model in 3F would indicate selective CST targeting of LPSNs expressing FGF22. What proportion of AAV-transduced non-LPSN spinal neurons are contacted by CST collaterals?

The arrowhead in 3G is in a different location in the separated channels than the merge. When combined with the overlapping colors, it makes it nearly impossible to determine where the red CST puncta is located. What does "DP" stand for in the y-axis label of 3G?

The use of variable y-axes in figure 4 behavioral plots makes it difficult to assess inter-experimental variability.

At what spinal level was the CST normalization performed?

Page 3, Line 92 - "We see a large number of LPSNs transduced..." Quantification in Figure 1D shows very few neurons are labeled. How do these numbers (~7-16) compare with the number labeled with fluorogold in the experiment from Figure 3?

Page 9 Line 407 - You state that FluoroGold was injected 9 days prior sacrifice. This contradicts the timeline in Figure 3A. Please reconcile.

Editing:
Figure 1A refers to hindlimb CST as hCST, yet the abbreviation is not used elsewhere or explained.

Figure 3F - The graph is missing "% Ctrl" on the y-axis label.
Caption Figure 3C - The upper panel is FGF, not control as stated.

Referee #2 (Comments on Novelty/Model System for Author):

Use of FGF22 to increase functional recovery after SCI is not new: Zhu et al 2020 (PMID: 32116697). It is surprising that this is not mentioned/discussed. This work is the follow-up to their previous publication (2015), and the novelty is moderate to low.

Referee #2 (Remarks for Author):

In this paper, the authors demonstrate that in vivo overexpression of FGF22 through viral transfer can enhance functional recovery following spinal cord injury, when delivered within 24 hours of injury. Further, they illustrate their claim by selectively targeting different neuronal populations to show that the transfer initiates enhanced circuit rewiring. Care was taken to define temporal constraints for application and ideas tested were completed in logical succession.
Overall, the presented manuscript was data driven, with clear illustrations of significant and convincing findings for most parts. Earlier research is used in a proper context for corroboration of their claims (although not mentioning Zhu et al 2020). Statistical analysis was performed appropriately and illustrated in multiple figures, with the methods section being clear and detailed regarding all performed tests and assessments. No further clarification would be needed to recreate the steps for corroboration by other researchers and all necessary materials are clearly defined.

Major and moderate concerns:
1. Use of FGF22 to increase functional recovery after SCI is not new: Zhu et al 2020 (PMID: 32116697). It is surprising that this is not mentioned/discussed. This work is the follow up to their previous publication (2015), and the novelty is moderate to low.
2. While the finding of a window opportunity for FGF22 treatment is interesting, the authors fall short at demonstrating the mechanisms. Indeed, it remains possible that remodeling still occurs when FGF22 is injected at 5days post SCI, and that the lack of recovery is due to the motoneurons death (motoneurons could be dying/already dead at that time). Therefore, the conclusion that remodeling is reduced with the delayed treatment is not supported by the present data. Proper characterization of this phenomenon, and impact on motoneuron death, is necessary to make that claim.
3. Functional recovery was assessed up to 21 dpi, but longer time point is required (>6 weeks) to demonstrate that the recovery is sustained. Additional behavior data (BMS, catwalk, as the authors previously used) would also further back the claim that broad targeting of neuronal populations with overexpression could eventually be translated into a clinical approach.
4. The discussion would benefit from a more in-depth emphasis on the significance and on the future from the results.
Minor comments:
Fig. 1B: top panel is confusing - it seems that the cortical AAV injections are performed before he cervical/lumbar (labeled 1-2-3 respectively) - but cervical/lumbar injections are made 6 days prior to cortical injections. Also, at the bottom of panel B- day 21 missing text? Also, consider switching the color for the lumbar from gray to orange to match the lumbar injection in the top panel
Fig. 3: Panel A misleading - BDA at 21days, but perfused 2 weeks after BDA injections, so anatomical analyzes occur at 35d, not 21. It is not clear for non-experts why that cervical levels are quantified, while but injections are performed using coordinates for hindlimbs (thoracic). Could the author comment on the potential impact of FGF22 on CST sprouting if cervical coordinates would be used?
Fig. 3C: the BDA tracing seems stronger in the FGF22 treated axons. The authors should discuss this. Could FGF2 impact the transport? Could this impact the quantifications of the boutons?
Fig. 4: for consistency, inverse the colors (FGF2 was orange in previous figures, this is confusing).
Overall:
While figures provided are well-designed and illustrate the claims, Figures 1G, 2C, and 3D-F could benefit from providing unmerged images, paired with final merged data, which is seen in Figures 1E and 3G and supplementary for improved clarity of results. Providing only the merged data makes it tedious to decipher between the different components being illustrated, especially in those where not much of one is present and multitudes of the other(s) are.
Improvements could be made in editing, with incorrect wording and misspellings scattered through the manuscript.

Referee #3 (Remarks for Author):

The manuscript by Aljovic et al. reported an effective approach to enhance circuit plasticity and functional recovery after partial spinal cord injury. Specifically, the study used AAV viral vectors to specifically express FGF22 in spinal cord excitatory neurons, which induced synaptogenesis and circuit rewiring, eventually formed relayed connections between injury corticospinal tract (CST) axon collateral branches and spinal cord motor neurons for functional recovery. Overall, partial spinal cord injury model more clinically relevant, and the study provided a more targeted approach to connect uninjured CST collateral branches to spinal cord interneurons and eventually to motor neurons. The use of FGF22 as the presynaptic organizing protein further improve the specificity of detour circuit formation. The weaknesses include the lack of mechanisms by which FGF22 mediating the targeted synaptogenesis, and the subsequent protective effects on motor neuron survival. It is also not clear why the treatment window exists. All the results were presented as number of contacts or synaptic boutons between CST collaterals and spinal cord interneurons. There was no direct evidence that such changes actually were responsible for the observed motor function recovery.

1. In Figure 1, by using 2 AAV viral vectors injected at different locations, the study achieved targeted expression of FGF22 in long propriospinal neurons (LPSN), which in turn promoted excitatory synapse formation between uninjured CST collaterals and LPSN axons. Presumably FGF22 expressed by LPSN was secreted out of infected LPSN neurons. If so, it is not clear why it only specifically induced synapse formation between CST collaterals and infected LPSN but other closely nearby LPSN?

2. In Figure 2, by using Cre dependent AAV viral vector encoding FGF22 and vGlu2-Cre mice, the study targeted FGF22 expression in all vGlu2 positive excitatory neurons in the spinal cord, which similarly induced increased contacts between uninjured CST collaterals and excitatory spinal cord interneurons. In addition, the study also observed increased spinal cord motor neurons survival with such more widely expression of FGF22, but not for more restricted expression in LPSN. However, how motor neurons survival was enhanced by one approach but not the other was unclear. Was it due to circuit-based neural activity indirectly or directly via the neurotrophic effects of FGF22?

3. In Figure 3, the study further expanded the expression of FGF22 to all spinal cord interneurons using AAV viral vector carrying the CMV promoter. It is well known that AAV infects not only neurons but also glial cells. The study needs to show some evidence that AAV used in the study specifically infected neurons.

4. In Figure 4, the study tested the functional recovery of using the pan-neuron infection approach, as well as the time window for the treatment. It was clear that the 2 motor behavior tests used showed significant improvement only for early treatments. However, there was no evidence provided if the observed functional recovery was indeed mediated by FGF22-induced spinal

cord circuit rewiring. Was enhanced motor neuron survival involved? Moreover, why did late treatment have no effects? Did such late fail to induce CST-spinal cord neuron synapse formation?

Additional comments from the referees:

Referee #3
In my opinion, the concerns mentioned could be addressed by additional experiments to a certain degree, which would significantly improve the quality of the manuscript. The previous study showed that FGF22 could improve recovery after SCI by inhibiting ER stress-induced neuronal cell death. If new experiments could in some degree validate that FGF22 enhances functional recovery after partial SCI by circuit rewiring, it is still novel in molecular mechanisms. To my knowledge, in the current SCI research field, many approaches could somehow enhance functional recovery, but without solid underlying mechanisms.

Referee #2
I agree that some of the concerns could be addressed (some of the new experiments may take a long time to be performed though), but I am concerned about the novelty and the fit to EMBO as it stands.

Referee #1
The Zhu 2020 findings do not impact the novelty of the approach in this manuscript. The survival effect proposed in that paper could be added to the discussion, but it is mechanistically distinct from the proposed mechanism of enhanced synaptogenesis. The distance between the AAV delivery and the lesion site lessens the possibility that some survival issue is at play here. Additionally, the Zhu paper used a distinct and non-standard injury model with limited evidence for uniform lesion size or reproducibility.

While I feel that the mechanistic questions remain, I am not convinced of the necessity to perform further longitudinal experiments to dissect out the contribution of the sprouting CST axons. The authors would need to address these shortcomings in a revised discussion section and reduce the implication that recovery is CST mediated. The 2004 Bareyre paper describing the sprouting of the injured hindlimb CST is one of the more highly cited SCI papers of its age, and there is a clear connection to those findings. However, the evidence for these connections in playing a role in functional recovery is absent and, as such, it should not be discussed in the results section.

For concerns over lumbar motor neuron survival, additional staining and quantification of tissue from the animals studied for behavior as well as appropriate controls and stereological methodology should be performed, or the section could be removed.

In my opinion, extending the experiment beyond 21 days is unnecessary. Even if there is a slight recovery over a longer time period, the difference is present over the first 3 weeks, when a large amount of recovery occurs. This is also the same period as in the prior EMBO publication, so I don't see any benefit to holding this to a different standard.

Point to point

EMM-2022-16111: Synaptogenic gene therapy with FGF22 improves circuit plasticity and functional recovery following spinal cord injury

:

Referee #1 (Remarks for Author):

Aljović and colleagues report on a molecular approach to enhance synapse remodeling after spinal cord injury. This work builds on their previous studies showing that corticospinal axons transected in the thoracic spinal cord sprout collaterals within the cervical cord and that the maturation of synaptic contacts made by these collaterals is dependent on fibroblast growth factor 22 (FGF22) signaling. Here they demonstrate that virally mediated FGF22 expression in the cervical spinal cord enhances maturation of novel contacts on long propriospinal neurons after thoracic injury. These propriospinal neurons have been established as critical drivers of hindlimb recovery after thoracic injury in rodent models. Furthermore, the authors find that viral transduction within the first day after injury can support recovery of hindlimb stepping on the irregular ladder test. The manuscript is well written, and the viral transduction and tracing methodology are well thought out. I have some concerns over interpretation of the findings and some missing methodological description, particularly as pertaining to poly-synaptic motor neuron loss. Overall, the study builds nicely on prior research output and provides a unique approach to enhancing formation of novel relay circuits after spinal cord injury. We thank the reviewer for her/his positive comments on our manuscript. We have answered below all comments raised by the reviewer.

The manuscript is largely focused on the role of the CST and corticospinal remodeling is implicated in the observed behavioral recovery. Surprisingly, no manipulations (optogenetic, chemogenetic, transection, conditional deletion of FGFR1R2, etc.) were made to test the CST contribution to recovery. The role for CST synaptic remodeling in functional recovery that is discussed in the results section is more speculative and would best be moved to the discussion. This is an important remark and we agree that showing the contribution of the hindlimb corticospinal tract (hCST) to the recovery pattern would be a good addition to the paper. As suggested by the reviewer, we have therefore used chemogenetics to specifically silence the hCST. To do so, we first selectively expressed the cre recombinase in long propriospinal neurons by injecting a retrograde AAV expressing cre into the lumbar spinal cord. Concomitantly, we systemically injected a PHP-eB viruses expressing FGF22 in a cre dependent way (DIO) so that long propriospinal neurons would specifically express FGF22. We then performed spinal cord injury and 14 days after the injury we delivered AAVs expressing silencing DREADD (AAV-hSyn-hM4Di-DREADD-mCherry) into layer 5 of the hindlimb cortex to specifically silence hindlimb CST neurons upon CNO delivery (see below and new **Expanded View Figure 4** in the revised paper). We tested the recovery of the mice on the irregular ladder rung and could demonstrate – in agreement with our previous experiments – that the mice do recover after FGF22 overexpression in propriospinal neurons. Following silencing of hCST neurons with CNO, we show a partial increase in mistakes in the ladder rung test indicating that the recovery is due – at least in part- to the remodeling of the hCST following FGF22 treatment (see below and new **Expanded View Figure 4B,C** in the revised paper). When we retested mice 24hrs following CNO injection, the number of mistakes decreased indicating at least a partial washout of the CNO over time (Rogers et al., 2021). The results of this experiment are added to the main manuscript as a new **Expanded View Figure**

4 as well as in lines 195 to 201 of the Results section, lines 459 to 465, 487 to 495 and 582 to 586 of the Material and Method section and lines 257 to 263 of the Discussion.

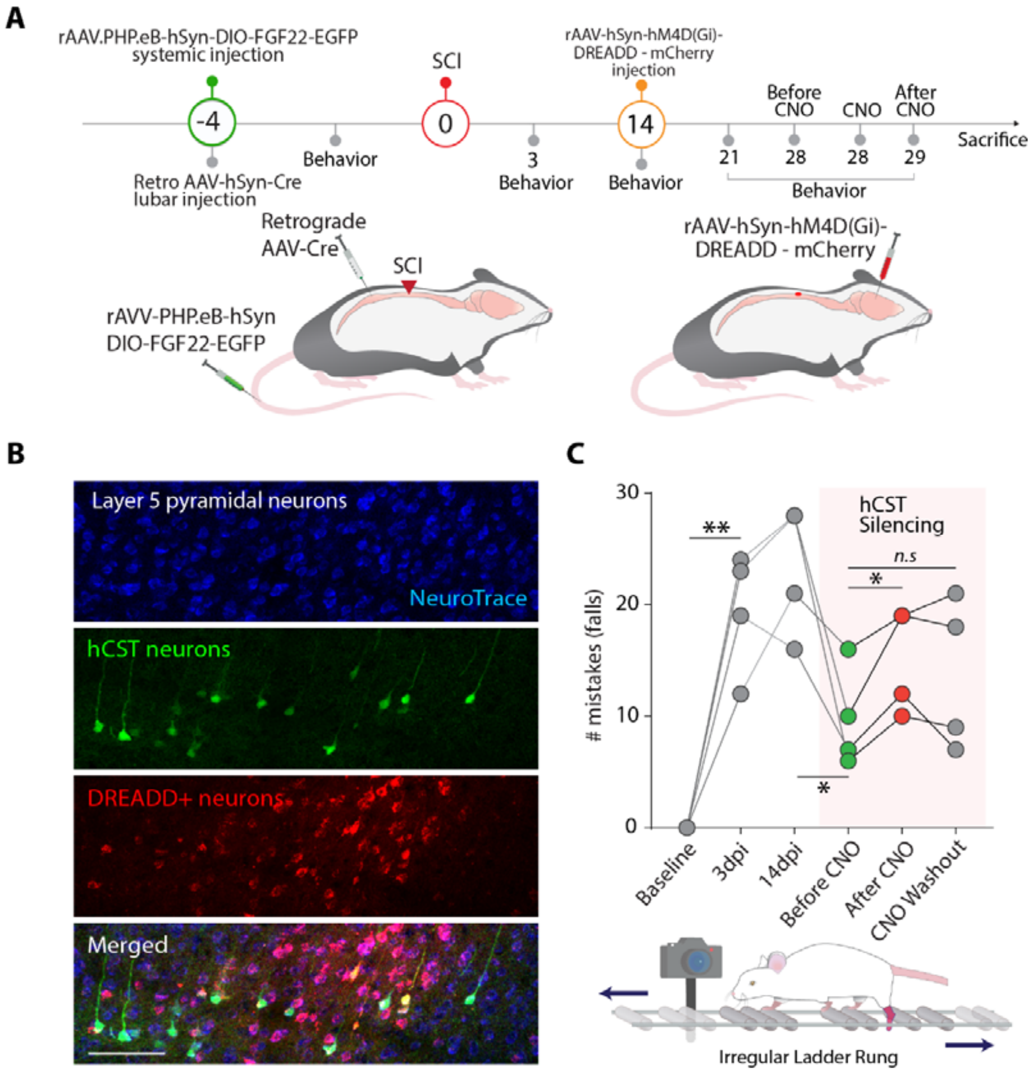

New Expanded View Figure 4. Chemogenetic silencing of the hCST demonstrates its contribution to functional recovery following spinal cord injury. (**A**) Time line of the experiment in which FGF22 is overexpressed in long propriospinal neurons and silencing DREADDs are delivered to the hindlimb cortex to specifically silence hCST neurons. (**B**) Confocal images of the DREADDs expressing hCST neurons retrogradely labeled with AAV-Cre (Neurotrace: blue; Retrogradely labeled hCST neurons: green; DREADD+ neurons: red). (**C**) Longitudinal quantifications of mistakes in the irregular ladder rung at baseline, 3days post injury (dpi), 14dpi, 28dpi before CNO administration (green dots), 28 dpi 30min after CNO administration (red dots) and 29dpi (CNO washout; grey dots) and scheme of the ladder rung task. Scale bar equals 100μm in B.

The proposed loss of lumbar motor neurons following thoracic injury is surprising. The degeneration described in the Han paper referenced (#43), and the study that it refers to, show no loss of lumbar motor neurons, but a dendritic atrophy and synaptic loss. What were the specific levels at which lumbar motor neurons were counted? Were stereological counts or corrections made to account for over or under sampling? What are the total projected numbers of motor neurons at these lumbar levels and do you have intact numbers of motor neurons in

the same area to demonstrate the extent of expected cell loss?
The motor neurons where counted at level L5. Stereological counts were not used at the time as we could sample for each section the entire spinal area containing motoneurons and have therefore counted all labeled neurons. However, in order to better quantify the motor neurons without over- or under-sampling we have now used unbiased deep learning models to quantify the number of motor neurons automatically. This model training and analysis was performed with the previously published U-Net toolbox (Falk et al., 2019). Interestingly we see a high degree of correlation of our previous count with the trained deep learning-based model (**Reviewer Figure 2A**).

In addition, in order to confirm that there is indeed motor neuron loss after spinal cord injury, we have now quantified the number of these neurons also in intact animals. We find that in control unlesioned animals the number of motoneurons is higher than in injured animal indicating a post-injury loss of motoneurons that is restored when FGF22 is delivered to all spinal cervical excitatory neurons (**Reviewer Figure 2** below and **Expanded View Figure 2**)

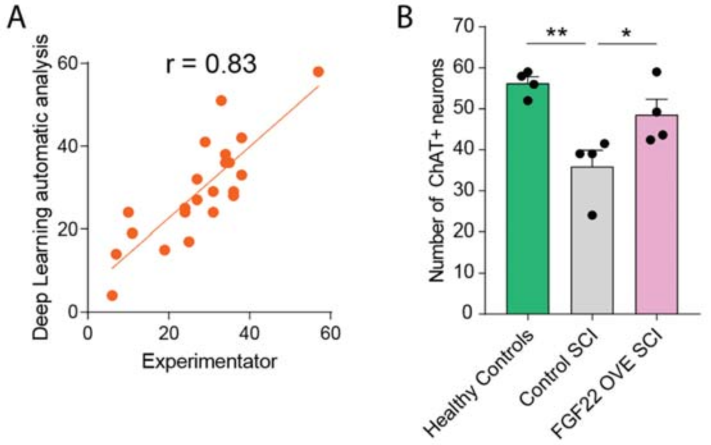

Reviewer figure 2: A - correlation between the manual count of motor neurons and the analysis using the deep–learning model U-Net**. B** – Quantification of motor neuron loss between healthy controls and injured animals overexpressing control or FGF22 virus.

We have thus added the motoneuron count in unlesioned mice, to the revised manuscript in Expanded View Figure 2 and discuss these data lines 307 and 314.

The authors propose that the similar recovery levels on regular and irregular ladder crossing with early AAV2/1-CMV-FGF22-IRES-GFP transduction may be due to distinct mechanisms on local versus descending circuit remodeling. It is more likely that these effects are related, with a reasonable interpretation being that supraspinal input enhances sensory-motor integration on the irregular ladder behavior. This behavior also raises the question of how does FGF22 over-expression affect presynaptic input from other populations of neurons (supraspinal, local, primary afferent)?
We agree with the reviewer that descending supraspinal input is likely to enhance sensory-motor integration and therefore affect both component of the ladder rung, regular and irregular. We have therefore revised our wording in the paper in particular lines 195-201. The other point raised by the reviewer is very interesting, namely that FGF22 is a general presynaptic organizer that can probably affect multiple descending supraspinal inputs. We fully agree with this assessment and apologize if this was not made clear enough in our initial writing. This is now better explained in the revised manuscript lines 300-306. In this context, it is interesting to note that the data obtained from the silencing experiment (see above) indicates that the recovery is

likely mediated only in part by the CST suggesting that other motor but also sensory tracts can probably contribute to the recovery process following treatment with FGF22. We discuss this points in lines 300-306 and lines 260-266.

The study on post-injury delivery of AAV2/1-CMV-FGF22-IRES-GFP nicely illustrates the time window for therapeutic intervention aimed at enhancing endogenous plasticity and circuit remodeling. How long after transduction are levels of FGF22 sufficient to induce synaptic maturation detected?
In line with a number of studies performed in vitro we believe that the action of FGF22 on the pre-synapse is quite rapid i.e. happens within few days. Indeed, in vitro after 6 days of overexpression one can already detect the effects of FGF22 on synaptic terminals (Terauchi et al., 2016). However, we believe that in our model of detour circuit formation, the action of FGF22 is primarily timed by the rewiring process that , as we demonstrated previously, shows the extension of the first hindlimb CST collaterals into the cervical cord from around 10d post-injury. Only after the collaterals have exited into the cervical cord can FGF22 act onto these to organize the presynapse on the hCST collaterals. This is likely between 14 and 21d after injury, a time frame that allows both an adequate amount of overexpressed FGF22 and its effects on synapses when delivered pre-injury but also at the acute and 1d delayed post-treatment time points. We have now further addressed this point in our Discussion lines 351-357 and cite the relevant literature.

What do numbers 1-4 mean in Figure 1B? They are not the order of procedures, as they are in 2A and 3A.
We apologize for the mistake. This is now corrected and the number represent indeed the order of the procedures.

Is the arrowhead in 1G meant to indicate a point of contact? If so, it doesn't appear to indicate a bouton-like pre-synaptic terminal.
We have now revised the panel and also present 3D views of the contacts made in Imaris from the deconvolved confocal image. The revised image panel and insets are found in Figure 1E and G. We also adapted the figure legend accordingly.

As the quantification in 1F indicates that only approximately 5-37% of boutons show vGlut1 staining, would you include a representative image of CST boutons without vGlut expression?
Following the Reviewer' suggestion, we have now added a representative image of CST boutons with and without vGlut expression. This can be found in the revised Figure 1E with insets showing boutons double labeled with vGlut or not. Those insets are also 3D views derived from the decomvolved confocal image.

Why do the normalized CST-LPSN contacts (1H) in controls show no variability, unlike other normalized control values?
In this experiment, the control group had a very low amount of contacts in comparison to the FGF22 treated group. Hence those animals all appear in a similar range after the normalization.

Are the number of samples in vGlut intensity plot in 1H indicating individual puncta? The context and interpretation of this data is not clear.
Each dot represents a long propriospinal neuron. Therefore, the graph shows the intensity of vGlut puncta on individual LPSNs in the control and FGF22 groups. We have made this clearer in the figure legend lines 827-828.

The truncated y-axis in Fig 3E prevents the interpretation of the control data. We have now changed the y-axis so that data can be better interpreted and visualized.

The model in 3F would indicate selective CST targeting of LPSNs expressing FGF22. What proportion of AAV-transduced non-LPSN spinal neurons are contacted by CST collaterals? The reviewer is right that our model was not well designed as also non LPSN transduced by FGF22 can be contacted by hCST collaterals as we showed in the Figure. We have revised the model accordingly. We have also quantified the percentage of non-LPSN interneurons contacted by hCST colalterals and found that it is ~ 18%.

The arrowhead in 3G is in a different location in the separated channels than the merge. When —combined with the overlapping colors, it makes it nearly impossible to determine where the red CST puncta is located. What does "DP" stand for in the y-axis label of 3G? We have now corrected the position of the arrowhead to allow visualization of the red puncta. DP stands for double positive. We have now added the explanation in the figure legend line 866.

The use of variable y-axes in figure 4 behavioral plots makes it difficult to assess inter-experimental variability. We have now adapted all y-axes in our paper to ease visualization of the effect and allow the assessment of inter experimental variability.

At what spinal level was the CST normalization performed? Long propriospinal neurons have their cell bodies at cervical levels C3 to C5. We thus performed all quantifications at those levels. We have now added this information in the Material and Methods lines 498.

Page 3, Line 92 - "We see a large number of LPSNs transduced..." Quantification in Figure 1D shows very few neurons are labeled. How do these numbers (~7-16) compare with the number labeled with fluorogold in the experiment from Figure 3? We label a similar amount of long propriosapinal neurons per section with any of our labeling techniques: about 10 to 15 as correctly pointed out by the Reviewer. Interestingly there is no major difference in number of labeled neurons between fluorogold and viral tracing. The main difference lies in the efficient labeling of long processes. With viral tracing, we can visualize long dendrites, while with fluorogold we label only the proximal dendrites. We have now added quantification of the number of labeled LPSNs to the a **revised Figure 3B** (see also below, **Reviewer Figure 3**). We have also changed the wording page 3 lines 101 to state the exact number of neurons labeled per section (13.04±2.8) and the figure legend of the **revised figure 3B** lines 848-850.

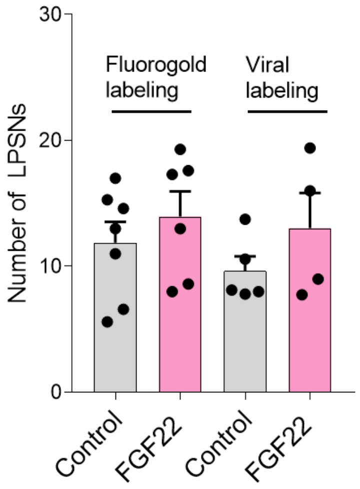

Reviewer Figure 3: Quantification of long propriospinal neurons labeled with either fluorogold or viral labeling in control (grey bars) and FGF22-transduced (pink bars) mice.

Page 9 Line 407 - You state that FluoroGold was injected 9 days prior sacrifice. This contradicts the timeline in Figure 3A. Please reconcile.
We apologize for the confusion. Fluorogold was indeed injected 9 days before sacrifice. We have now corrected the timeline in Figure 3A.

Editing:
Figure 1A refers to hindlimb CST as hCST, yet the abbreviation is not used elsewhere or explained.
Thanks. We have now explained the abbreviation in the text of the introduction line 55 and in the figure 1 legend lines 813-814.

Figure 3F - The graph is missing "% Ctrl" on the y-axis label.
Caption Figure 3C - The upper panel is FGF, not control as stated.
Thanks. This is now corrected.

Referee #2 (Remarks for Author):

In this paper, the authors demonstrate that in vivo overexpression of FGF22 through viral transfer can enhance functional recovery following spinal cord injury, when delivered within 24 hours of injury. Further, they illustrate their claim by selectively targeting different neuronal populations to show that the transfer initiates enhanced circuit rewiring. Care was taken to define temporal constraints for application and ideas tested were completed in logical succession.
Overall, the presented manuscript was data driven, with clear illustrations of significant and convincing findings for most parts. Earlier research is used in a proper context for corroboration of their claims (although not mentioning Zhu et al 2020). Statistical analysis was performed appropriately and illustrated in multiple figures, with the methods section being clear and detailed regarding all performed tests and assessments. No further clarification would be needed to recreate the steps for corroboration by other researchers and all necessary materials are clearly defined.

We thank the reviewer for appreciating the scientific quality of our work.

Major and moderate concerns:
1. Use of FGF22 to increase functional recovery after SCI is not new: Zhu et al 2020 (PMID: 32116697). It is surprising that this is not mentioned/discussed. This work is the follow up to their previous publication (2015), and the novelty is moderate to low.

We apologize for omitting the important work of Zhu et al. This is now corrected in the revised manuscript. We believe that while we established the role of FGF22 as an endogenous synaptogenic modulator following SCI in our previous work (Jacobi et al., 2015), its use as a synaptogenic therapeutic agent that can improve the recovery from spinal cord injury is new. Furthermore we show that this therapeutic effect is mediated by the effects of FGF22 on circuit plasticity which is both a mechanistically distinct process and a distinct therapeutic target compared to the anti-apoptotic effects reported by Zhu et al. after local injection of FGF 22 at the lesion site. We thus believe that our findings are both novel and exciting as circuit remodeling has been shown to be critical for long term recovery following spinal cord injury (Bareyre et al., 2004; Bradley et al.,2019).

In order to better relate our work to the elegant studies by Zhu et al., we have now determined the size of the spinal cord lesions in control and FGF22 treated mice. In contrast to Zhu et al., we cannot find any differences in lesion volume (see below). This suggests that the FGF22 overexpression in our experiments is induced too far from the lesion site to affect local lesion processes e.g. through an anti-apoptotic mechanism See below **Reviewer Figure 4**). We discuss our findings as complementary to those of Zhu et al., in the revised discussion of the paper lines 307-314.

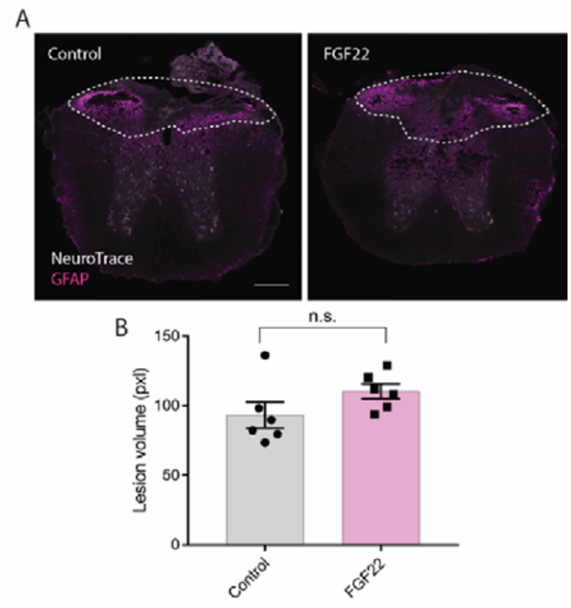

Reviewer Figure 4: A- Confocal images of the T8 hemisection in control and FGF22-treated mice. B- Quantification of lesion volume in control (grey bar) and FGF22-treated mice (purple bar). Scale bar equals 200µm.

.

2. While the finding of a window opportunity for FGF22 treatment is interesting, the authors fall short at demonstrating the mechanisms. Indeed, it remains possible that remodeling still occurs when FGF22 is injected at 5days post SCI, and that the lack of recovery is due to the motoneurons death (motoneurons could be dying/already dead at that time). Therefore, the conclusion that remodeling is reduced with the delayed treatment is not supported by the present

data. Proper characterization of this phenomenon, and impact on motoneuron death, is necessary to make that claim.

We agree with the reviewer that the motoneuron death could be different at acute and 5d post-injury. In order to remove this uncertainty, we have now quantified the number of motoneurons at lumbar levels L1 at acute and 5d post-injury between control- and FGF22-treated mice. We could see no difference between groups neither at the acute nor at the 5d time point (see below **Reviewer Figure 5**). This indicates that the same number of motoneurons are present 3 weeks following injury in all groups ruling out an early protection of motoneurons with the acute delivery of FGF22. This therefore strengthen our interpretation that there is a therapeutic window of treatment for FGF22 that can induce synapse maturation during post-injury circuit rewiring.

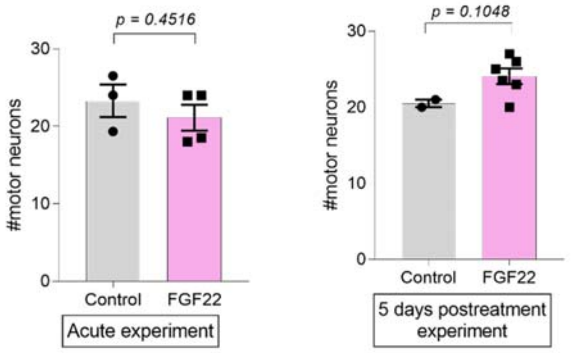

Reviewer Figure 5: Quantification of motoneurons are lumbar level L1 in control (grey bars) and FGF22-treated (pink bars) mice, following acute treatment with FGF22 (**Left**) or delayed treatment at 5d (**Right**).

3. Functional recovery was assessed up to 21 dpi, but longer time point is required (>6 weeks) to demonstrate that the recovery is sustained. Additional behavior data (BMS, catwalk, as the authors previously used) would also further back the claim that broad targeting of neuronal populations with overexpression could eventually be translated into a clinical approach.

We thank the reviewer for the suggestion. However based on the discussion between reviewers during the pre-decision cross-commenting process (also provided to us by the editor) we understood that these particularly time-demanding experiments are outside the scope of the current study.

4. The discussion would benefit from a more in-depth emphasis on the significance and on the future from the results.

We thank the reviewer for their suggestion and have done so lines 351-357 and 369-374.

Minor comments:

Fig. 1B: top panel is confusing - it seems that the cortical AAV injections are performed before he cervical/lumbar (labeled 1-2-3 respectively) - but cervical/lumbar injections are made 6 days prior to cortical injections. Also, at the bottom of panel B- day 21 missing text? Also, consider switching the color for the lumbar from gray to orange to match the lumbar injection in the top panel

We thank the reviewer for this suggestion and apologize for the mislabeling. We now have changed Fig.1B top panel.

Fig. 3: Panel A misleading - BDA at 21days, but perfused 2 weeks after BDA injections, so anatomical analyzes occur at 35d, not 21. It is not clear for non-experts why that cervical levels

are quantified, while but injections are performed using coordinates for hindlimbs (thoracic). Could the author comment on the potential impact of FGF22 on CST sprouting if cervical coordinates would be used?

We apologize as we had forgotten to put the BDA injection on the timeline. As BDA was injected two weeks before sacrifice, the analysis are also performed at 21d as in the rest of the paper. We have now corrected this.

We agree that the paradigm of detour circuit formation during which collaterals from the hindlimb CST (hCST) remodel in the cervical cord (Bareyre et al., 2004; Lang et al., 2012; Jacobi et al,. 2015; Bradley et al., 2019) could be better explained and have done so in the revised manuscript lines 54,55 and lines 87. Likewise we are now addressing the possible impact of FGF22 on forelimb CST collaterals in the revised discussion 291-293.

Fig. 3C: the BDA tracing seems stronger in the FGF22 treated axons. The authors should discuss this. Could FGF2 impact the transport? Could this impact the quantifications of the boutons?

As the reviewer suggested we quantified the number of CST axons in the dorsal column to control for the labeling efficiency as well as the intensity of CST collaterals within the grey matter to determine whether the labeling has an impact on axonal transport. Although there is some variation in labeling (as expected with such anatomical tracing), we could not detect any overall differences between the groups likely indicating that FGF22 does not overtly change transport (see below, **Reviewer Figure 6**).

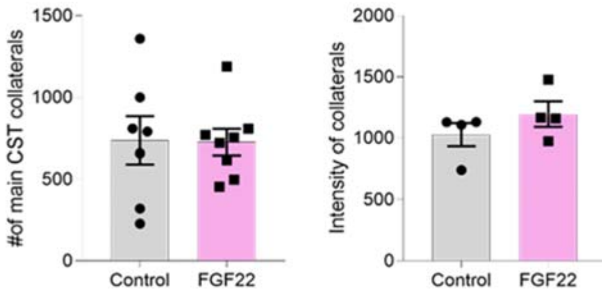

Reviewer figure 6: Quantification of CST axons and collaterals in control mice and mice treated with FGF22. A- Quantification of the number of CST axons in the dorsal column and B- quantification of the intensity of CST collaterals in the cervical spinal cord.

Fig. 4: for consistency, inverse the colors (FGF2 was orange in previous figures, this is confusing).

We apologize for the confusion and have done so.

Overall:

While figures provided are well-designed and illustrate the claims, Figures 1G, 2C, and 3D-F could benefit from providing unmerged images, paired with final merged data, which is seen in Figures 1E and 3G and supplementary for improved clarity of results. Providing only the merged data makes it tedious to decipher between the different components being illustrated, especially in those where not much of one is present and multitudes of the other(s) are. Improvements could be made in editing, with incorrect wording and misspellings scattered through the manuscript.

We understand that providing unmerged images would strengthen the manuscript. We therefore thank the reviewer for the suggestion and have done so.

Referee #3 (Remarks for Author):

The manuscript by Aljovic et al. reported an effective approach to enhance circuit plasticity and functional recovery after partial spinal cord injury. Specifically, the study used AAV viral vectors to specifically express FGF22 in spinal cord excitatory neurons, which induced synaptogenesis and circuit rewiring, eventually formed relayed connections between injury corticospinal tract (CST) axon collateral branches and spinal cord motor neurons for functional recovery. Overall, partial spinal cord injury model more clinically relevant, and the study provided a more targeted approach to connect uninjured CST collateral branches to spinal cord interneurons and eventually to motor neurons. The use of FGF22 as the presynaptic organizing protein further improve the specificity of detour circuit formation. The weaknesses include the lack of mechanisms by which FGF22 mediating the targeted synaptogenesis, and the subsequent protective effects on motor neuron survival. It is also not clear why the treatment window exists. All the results were presented as number of contacts or synaptic boutons between CST collaterals and spinal cord interneurons. There was no direct evidence that such changes actually were responsible for the observed motor function recovery.
We thank the reviewer for her/his assessment of our work and have addressed all remaining concerns below.

1. In Figure 1, by using 2 AAV viral vectors injected at different locations, the study achieved targeted expression of FGF22 in long propriospinal neurons (LPSN), which in turn promoted excitatory synapse formation between uninjured CST collaterals and LPSN axons. Presumably FGF22 expressed by LPSN was secreted out of infected LPSN neurons. If so, it is not clear why it only specifically induced synapse formation between CST collaterals and infected LPSN but other closely nearby LPSN?
We believe that the Reviewer refers to the data in Figure 3 that indeed shows increased CST contacts on LPSN transduced by FGF22 and not on those nearby. This to us indicates that while FGF22 is secreted, the diffusion into the tissue is likely limited (possibly by binding to the extracellular matrix) resulting in the existence of tissue gradients as well known e.g. for axon guidance molecules (Chédotal et al., 2019). We now better discuss these findings in the revised manuscript 291-294.

2. In Figure 2, by using Cre dependent AAV viral vector encoding FGF22 and vGlu2-Cre mice, the study targeted FGF22 expression in all vGlu2 positive excitatory neurons in the spinal cord, which similarly induced increased contacts between uninjured CST collaterals and excitatory spinal cord interneurons. In addition, the study also observed increased spinal cord motor neurons survival with such more widely expression of FGF22, but not for more restricted expression in LPSN. However, how motor neurons survival was enhanced by one approach but not the other was unclear. Was it due to circuit-based neural activity indirectly or directly via the neurotrophic effects of FGF22?

To assess direct cyto-protective effects of FGF22 we quantified the lesion volume and did not detect a difference between the FGF22 and the control group (**see also Comment to Reviewer 2 and Reviewer Figure 4 above**). This would suggest that viral gene transfer of FGF22 to the cervical spinal cord cannot prevent apoptosis in the thoracic cord (while apoptosis was

prevented e.g. by Zhu et al via direct injection of FGF22 at the injury site (Zhu et al., 2020). As the lumbar cord is even further distant to the cervical cord, this would make it likely that the effects of FGFG22 on motoneurons survival are more likely related to the indirect actions of FGF22 e.g. on circuit structure and activity. This is however of course difficult to prove.
The action of FGF22 on motoneurons is discussed lines 307-314.

3. In Figure 3, the study further expanded the expression of FGF22 to all spinal cord interneurons using AAV viral vector carrying the CMV promoter. It is well known that AAV infects not only neurons but also glial cells. The study needs to show some evidence that AAV used in the study specifically infected neurons.
The reviewer is correct in pointing out that the CMV promoter does not induce expression only in neurons but can also transduce other cell types in particular in glial cells. To better characterize the transduction pattern, we have now quantified the percentage of neurons transduced in those animals and can see that on average about a third of the transduced cells in these experiments are neurons. To better reflect the broad targeting of neurons and glial cells in this set of experiments we now refer to the application strategy as "Non-selective" throughout the paper to better differentiate from the selective neuronal targeting strategies employed in the rest of the study (lines 165-168 in the Results section). We also discuss the limitations of this particular targeting strategy in the revised discussion lines 233-237 and include the quantification of the transduction pattern in the revised Methods lines 487-495.

4. In Figure 4, the study tested the functional recovery of using the pan-neuron infection approach, as well as the time window for the treatment. It was clear that the 2 motor behavior tests used showed significant improvement only for early treatments. However, there was no evidence provided if the observed functional recovery was indeed mediated by FGF22-induced spinal cord circuit rewiring. Was enhanced motor neuron survival involved? Moreover, why did late treatment have no effects? Did such late fail to induce CST-spinal cord neuron synapse formation?

The reviewer asks us to further investigate the anatomical basis of the improved functional recovery observed in response to "therapeutic" FGF22 delivery at 1 day post injury. We have performed two experiments to directly address this:

First, to control for a different motoneuron survival in acute delivery of FGF22 post-injury and delayed delivery till 5days post-injury, we quantified the number of motoneurons in the lumbar cord. We could see no difference between groups neither at the acute nor at the 5d time point see also **Comment to Reviewer 2** and **Reviewer Figure 5**). This indicates that the same number of motoneurons are present 3 weeks following injury in all groups ruling out an early protection of motoneurons with the acute delivery of FGF22.

Second, to verify that the FGF22 effects on corticospinal remodeling are present both when FGF22 is delivered pre-injury are also recapitulated when FGF22 is delivered 1 day after injury and that the behavioral effects are also triggered by FGF22 we have performed immunohistochemistry on those animals treated with FGF22 post-injury (24hrs group). As FGF22 has been shown to induce presynaptic differentiation including clustering of synaptic vesicles, formation of active zones, and cytoskeletal restructuring and assembly of vesicle recycling machinery (Umemori et al., 2004), we investigated the expression of bassoon, an active zone marker and the synaptic vesicle-associated protein synapsin. In line with our

previous results, we demonstrate that the post-injury overexpression of FGF22 in spinal neurons enhances the maturation of presynaptic boutons along the CST collaterals as more boutons expressed synapsin and bassoon (see below and **new revised Fig.4 F,G** in the revised paper). This indicates that when FGF22 is delivered after the injury, it also organizes the pre-synapses and triggers the maturations of boutons along the presynaptic hCST collaterals. We have now added those data to our revised paper on the **new revised Figure 4 F,G** as well as in the results lines 207-212 and discussion lines 276-278.

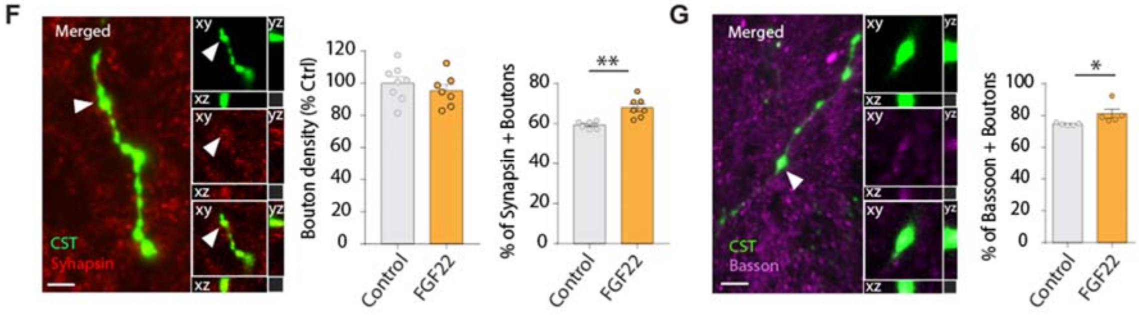

New figure panel Fig. 4. (F) Representative confocal image of CST boutons and synapsin staining. and quantification of the percentage of bouton synapsin positive (n = 6-7 mice per group). (G) Representative confocal images of CST bouton and bassoon staining and quantification of the percentage of boutons that are bassoon positive (n = 5-6 mice per group). Insets in F and G represent 3D views generated in Imaris of the confocal images. Scale bars equals 10µm in F,G.

References

Falk, T., Mai, D., Bensch, R. et al. U-Net: deep learning for cell counting, detection, and morphometry. Nat Methods 16, 67–70 (2019).

Rogers S, Rozman PA, Valero M, Doyle WK, Buzsáki G. Mechanisms and plasticity of chemogenically induced interneuronal suppression of principal cells. Proc Natl Acad Sci U S A. 2021 Jan 12;118(2):e2014157118.

Terauchi A, Johnson-Venkatesh EM, Bullock B, Lehtinen MK, Umemori H. Retrograde fibroblast growth factor 22 (FGF22) signaling regulates insulin-like growth factor 2 (IGF2) expression for activity-dependent synapse stabilization in the mammalian brain. Elife. 2016 Apr 15;5:e12151.

Jacobi A, Loy K, Schmalz AM, Hellsten M, Umemori H, Kerschensteiner M, Bareyre FM. FGF22 signaling regulates synapse formation during post-injury remodeling of the spinal cord. EMBO J. 2015 May 5;34(9):1231-43.

Lang C, Guo X, Kerschensteiner M, Bareyre FM. Single collateral reconstructions reveal distinct phases of corticospinal remodeling after spinal cord injury. PLoS One. 2012;7(1):e30461.

Bareyre FM, Kerschensteiner M, Raineteau O, Mettenleiter TC, Weinmann O, Schwab ME. The injured spinal cord spontaneously forms a new intraspinal circuit in adult rats. Nat Neurosci. 2004 Mar;7(3):269-77.

Bradley PM, Denecke CK, Aljovic A, Schmalz A, Kerschensteiner M, Bareyre FM. Corticospinal circuit remodeling after central nervous system injury is dependent on neuronal activity. J Exp Med. 2019 Nov 4;216(11):2503-2514.

Zhu S, Chen M, Chen M, Ye J, Ying Y, Wu Q, Dou H, Bai L, Mao F, Ni W, Yu K. Fibroblast Growth Factor 22 Inhibits ER Stress-Induced Apoptosis and Improves Recovery of Spinal Cord Injury. Front Pharmacol. 2020 Feb 11;11:18.

Chédotal A. Roles of axon guidance molecules in neuronal wiring in the developing spinal cord. Nat Rev Neurosci. 2019 Jul;20(7):380-396.

1st Revision - Editorial Decision — 1st Dec 2022

Thank you for the submission of your revised manuscript to EMBO Molecular Medicine. We have now received the enclosed report from the two referees who were asked to re-assess it. As you will see, the referees are overall supportive, and I am pleased to inform you that we will be able to accept your manuscript pending the following amendments:

1. Please address the remaining concerns of Referee #1 in writing, especially Point #1 regarding the statement about CST's role in functional recovery.

On a more editorial level:

**** Reviewer's comments *****

Referee #1 (Remarks for Author):

The authors have largely addressed the concerns of the previous review; however, the statements regarding hCST underlying functional recovery need to be revised.

While it was not specifically requested, the authors performed a silencing experiment to test the necessity for hindlimb CST contributions in functional recovery. The experiment as performed; however, does not directly test the CST contribution to behavioral recovery, but rather shows a mild effect of silencing hindlimb cortex with DREADDs on the irregular ladder cross behavior. As such, the interpretation is not supported fully by the experimental results and the text should be edited to indicate that there is a role for the motor cortex rather than hCST specifically. I would reiterate that the role for hCST synaptic remodeling in functional recovery is still somewhat speculative and statements regarding this should therefore be removed from the results and those in the discussion should be revised.

From the image of cortical transduction provided (Exp View Fig 4B), there is transduction of multiple populations of hindlimb motor cortex neurons and it is not clear what proportion of hindlimb CST neurons expresses the inhibitory DREADD hM4D(Gi). Nor is it clear why systemic injection of AAV.PHP.eB-hSyn-DIO-FGF22-EGFP was used to transduce a widespread population of both spinal neurons and retrogradely transduced CST neurons. As retrograde AAV-Cre was injected to the spinal cord, it was not clear what the rationale was for not using Cre-dependent vectors to selectively express hM4D(Gi) in hCST neurons as well as FGF22 in LPSNs, as in Figure 1.

Please use the same y-axis scale on Exp View Fig 4c as in Fig 4c

Title of Ex View Fig 4 should be changed to, "Chemogenetic silencing of hindlimb motor cortex demonstrates its contribution to functional recovery following spinal cord injury." The results do not indicate a role for hCST neurons, but rather show that silencing hindlimb M1 mildly impairs irregular ladder crossing.

Referee #3 (Remarks for Author):

The revised manuscript addressed most of my comments with either more discussion or new experimental results. In particular, the new chemogenetic silencing experiment greatly improve the supporting evidence for the main conclusion of the study. The study provided a new approach for spinal cord regeneration and the subsequent functional recovery.

Point to point EMM-2022-16111-V2

Referee #1 (Remarks for Author):

The authors have largely addressed the concerns of the previous review; however, the statements regarding hCST underlying functional recovery need to be revised.

While it was not specifically requested, the authors performed a silencing experiment to test the necessity for hindlimb CST contributions in functional recovery. The experiment as performed; however, does not directly test the CST contribution to behavioral recovery, but rather shows a mild effect of silencing hindlimb cortex with DREADDs on the irregular ladder cross behavior. As such, the interpretation is not supported fully by the experimental results and the text should be edited to indicate that there is a role for the motor cortex rather than hCST specifically. I would reiterate that the role for hCST synaptic remodeling in functional recovery is still somewhat speculative and statements regarding this should therefore be removed from the results and those in the discussion should be revised.

From the image of cortical transduction provided (Exp View Fig 4B), there is transduction of multiple populations of hindlimb motor cortex neurons and it is not clear what proportion of hindlimb CST neurons expresses the inhibitory DREADD hM4D(Gi). Nor is it clear why systemic injection of AAV.PHP.eB-hSyn-DIO-FGF22-EGFP was used to transduce a widespread population of both spinal neurons and retrogradely transduced CST neurons. As retrograde AAV-Cre was injected to the spinal cord, it was not clear what the rationale was for not using Cre-dependent vectors to selectively express hM4D(Gi) in hCST neurons as well as FGF22 in LPSNs, as in Figure 1.

Please use the same y-axis scale on Exp View Fig 4c as in Fig 4c

Title of Ex View Fig 4 should be changed to, "Chemogenetic silencing of hindlimb motor cortex demonstrates its contribution to functional recovery following spinal cord injury." The results do not indicate a role for hCST neurons, but rather show that silencing hindlimb M1 mildly impairs irregular ladder crossing.

We have followed the reviewer's suggestion and changed our wording in the paper to implicate the hindlimb motor cortex rather than the hCST in the recovery. The changes can be found throughout the results and discussion.

In this experiment we used the AAV.PHP.eB-hSyn-DIO-FGF22-EGFP systemic injection in order to increase the number of propriospinal neurons infected and obtain a behavioral readout. We reasoned that with a PHP.eB virus and a systemic delivery we would probably target substantially more LPSN than with a local injection as this would target several segments of the cervical cord. For the silencing we used a cre-dependent construct (AAV8- hSyn-hM4D(Gi)-DREADD-mCherry) to locally silence the hindlimb motor cortex. We have now added a sentence to explain the use of the AAV.PHP.eB-hSyn-DIO-FGF22-EGFP in the silencing experiment lines 459-461 of the paper.

We have now used the same y-axis in Exp View Fig.4c as in Fig.4c.

We have now changed the title of Exp View Fig4 as suggested by the reviewer to emphasize the role of the hindlimb motor cortex rather than hCST.

Referee #3 (Remarks for Author):

The revised manuscript addressed most of my comments with either more discussion or new experimental results. In particular, the new chemogenetic silencing experiment greatly improve the supporting evidence for the main conclusion of the study. The study provided a new approach for spinal cord regeneration and the subsequent functional recovery.

We thank the reviewer for her/his comments on our manuscript.

2nd Revision - Editorial Decision 15th Dec 2022

I am pleased to inform you that your manuscript is accepted for publication and is now being sent to our publisher to be included in the next available issue of EMBO Molecular Medicine.

EMBO Press Author Checklist

Corresponding Author Name: Florence M Bareyre	
Journal Submitted to: EMBO Molecular Medicine	
Manuscript Number: EMM-2022-16111	

USEFUL LINKS FOR COMPLETING THIS FORM
The EMBO Journal - Author Guidelines
EMBO Reports - Author Guidelines
Molecular Systems Biology - Author Guidelines
EMBO Molecular Medicine - Author Guidelines

Reporting Checklist for Life Science Articles (updated January
This checklist is adapted from Materials Design Analysis Reporting (MDAR) Checklist for Authors. MDAR establishes a minimum set of requirements in transparent reporting in the life sciences (see Statement of Task: 10.31222/osf.io/9sm4x). Please follow the journal's guidelines in preparing your
Please note that a copy of this checklist will be published alongside your article.

Abridged guidelines for figures
1. Data
The data shown in figures should satisfy the following conditions:
- the data were obtained and processed according to the field’s best practice and are presented to reflect the results of the experiments in an accurate and unbiased manner.
- ideally, figure panels should include only measurements that are directly comparable to each other and obtained with the same assay.
- plots include clearly labeled error bars for independent experiments and sample sizes. Unless justified, error bars should not be shown for technical
- if n<5, the individual data points from each experiment should be plotted. Any statistical test employed should be justified.
- Source Data should be included to report the data underlying figures according to the guidelines set out in the authorship guidelines on Data

2. Captions
Each figure caption should contain the following information, for each panel where they are relevant:
- a specification of the experimental system investigated (eg cell line, species name).
- the assay(s) and method(s) used to carry out the reported observations and measurements.
- an explicit mention of the biological and chemical entity(ies) that are being measured.
- an explicit mention of the biological and chemical entity(ies) that are altered/varied/perturbed in a controlled manner.
- the exact sample size (n) for each experimental group/condition, given as a number, not a range;
- a description of the sample collection allowing the reader to understand whether the samples represent technical or biological replicates (including how many animals, litters, cultures, etc.).
- a statement of how many times the experiment shown was independently replicated in the laboratory.
- definitions of statistical methods and measures:
  - common tests, such as t-test (please specify whether paired vs. unpaired), simple χ2 tests, Wilcoxon and Mann-Whitney tests, can be unambiguously identified by name only, but more complex techniques should be described in the methods section;
  - are tests one-sided or two-sided?
  - are there adjustments for multiple comparisons?
  - exact statistical test results, e.g., P values = x but not P values < x;
  - definition of ‘center values’ as median or average;
  - definition of error bars as s.d. or s.e.m.

Please complete ALL of the questions below.
Select "Not Applicable" only when the requested information is not relevant for your study.

Materials

Newly Created Materials	Information included in the manuscript?	In which section is the information available? (Reagents and Tools Table, Materials and Methods, Figures, Data Availability Section)
New materials and reagents need to be available; do any restrictions apply?	Yes	Any material or tools used in this paper will be made available to scientists. This included FGF22 plasmids. Information can be found in the Material and Methods section

Antibodies	Information included in the manuscript?	In which section is the information available? (Reagents and Tools Table, Materials and Methods, Figures, Data Availability Section)
For **antibodies** provide the following information:<br>- Commercial antibodies: RRID (if possible) or supplier name, catalogue number and or/clone number<br>- Non-commercial: RRID or citation	Yes	Anti-GFP antibody (#A11122; Life Technologies, 1:500). Goat Anti-Rabbit-488 secondary antibody (#A11070 Life Technologies; 1:500). Rabbit anti-vGlut1 (1:500; Synaptic Systems, #135303). Rabbit anti-vGlut1/2 (1:500; Synaptic Systems, #135503). Guinea pig anti-vGAT (1:1000; Synaptic Systems, #131308). Rabbit anti-Synapsin (1:500; Millipore). Mouse anti-

DNA and RNA sequences	Information included in the manuscript?	In which section is the information available? (Reagents and Tools Table, Materials and Methods, Figures, Data Availability Section)
**Short novel DNA or RNA including primers, probes**: provide the sequences.	Yes	Primer sequences and original plasmid used to clone FGF22 are provided. No other new sequences. Only available plasmids were used to clone

Cell materials	Information included in the manuscript?	In which section is the information available? (Reagents and Tools Table, Materials and Methods, Figures, Data Availability Section)
**Cell lines:** Provide species information, strain. Provide accession number in repository **OR** supplier name, catalog number, clone number, and/**OR** RRID.	Not Applicable	
**Primary cultures:** Provide species, strain, sex of origin, genetic modification status.	Yes	This is provided in the Material and Methods section.
Report if the cell lines were recently **authenticated** (e.g., by STR profiling) and tested for mycoplasma contamination.	Not Applicable	

Experimental animals	Information included in the manuscript?	In which section is the information available? (Reagents and Tools Table, Materials and Methods, Figures, Data Availability Section)
**Laboratory animals or Model organisms:** Provide species, strain, sex, age, genetic modification status. Provide accession number in repository **OR** supplier name, catalog number, clone number, **OR** RRID.	Yes	provided in the Material and Method section under headline "Mice". We used adult female C57Bl/6j and adult female VGlut2-Cre (RRID:IMSR_JAX:016963) mice aged of 8-12 weeks at the start of the experiment.
**Animal observed in or captured from the field:** Provide species, sex, and age where possible**.**	Not Applicable	
Please detail **housing and husbandry conditions**.	Yes	Done in the Material and Method section. Mice were maintained on a 12h light/ 12h dark cycle with food and water ad libitum.

Plants and microbes	Information included in the manuscript?	In which section is the information available? (Reagents and Tools Table, Materials and Methods, Figures, Data Availability Section)
**Plants:** provide species and strain, ecotype and cultivar where relevant, unique accession number if available, and source (including location for collected wild specimens).	Not Applicable	
**Microbes:** provide species and strain, unique accession number if available, and source.	Not Applicable	

Human research participants	Information included in the manuscript?	In which section is the information available? (Reagents and Tools Table, Materials and Methods, Figures, Data Availability Section)
If collected and within the bounds of privacy constraints report on age, sex and gender or ethnicity for all study participants.	Not Applicable	

Core facilities	Information included in the manuscript?	In which section is the information available? (Reagents and Tools Table, Materials and Methods, Figures, Data Availability Section)
If your work benefited from core facilities, was their service mentioned in the acknowledgments section?	Yes	We used the Core Bioimaging Facility of the Biomedical Center. Statement can be found in the acknowledgment section

Design

Study protocol	Information included in the manuscript?	In which section is the information available? (Reagents and Tools Table, Materials and Methods, Figures, Data Availability Section)
If study protocol has been **pre-registered, provide DOI in the manuscript**. For clinical trials, provide the trial registration number **OR** cite DOI.	Not Applicable	
Report the **clinical trial registration number** (at ClinicalTrials.gov or equivalent), where applicable.	Not Applicable	

Laboratory protocol	Information included in the manuscript?	In which section is the information available? (Reagents and Tools Table, Materials and Methods, Figures, Data Availability Section)
Provide DOI OR other citation details if **external detailed step-by-step protocols** are available.	Not Applicable	

Experimental study design and statistics	Information included in the manuscript?	In which section is the information available? (Reagents and Tools Table, Materials and Methods, Figures, Data Availability Section)
Include a statement about **sample size** estimate even if no statistical methods were used.	Yes	No sample size estimate was performed. A statement has been added.
Were any steps taken to minimize the effects of subjective bias when allocating animals/samples to treatment (e.g. **randomization procedure**)? If yes, have they been described?	Yes	Animals were randomly assigned to treatments by a person blinded to the injury or treatment status whenever possible.
Include a statement about **blinding** even if no blinding was done.	Yes	All analysis were performed by blinded observers. A statement is present in the Material and Methods section.
Describe **inclusion/exclusion criteria** if samples or animals were excluded from the analysis. Were the criteria pre-established?<br><br>If sample or data points were omitted from analysis, report if this was due to attrition or intentional exclusion and provide justification.	Yes	No data were excluded from the study. The only occurence when mice were not included is if the lesion was poorly performed or in case of poor labeling of the hindlimb CST or LPSN.
For every figure, are **statistical tests** justified as appropriate? Do the data meet the assumptions of the tests (e.g., normal distribution)? Describe any methods used to assess it. Is there an estimate of variation within each group of data? Is the variance similar between the groups that are being statistically compared?	Yes	For every statistical tests we first tested for normality with a d'Agostino-pearson omnibus normlaity test and then performed the appropriate statistical tests. All tests are described in the Material and Methods section. In all statistics we assumed similar variances.

Sample definition and in-laboratory replication	Information included in the manuscript?	In which section is the information available? (Reagents and Tools Table, Materials and Methods, Figures, Data Availability Section)
In the figure legends: state number of times the experiment was **replicated** in laboratory.	Yes	This is done in all figure legends
In the figure legends: define whether data describe **technical or biological replicates**.	Yes	This is done in all figure legends

Ethics

Ethics	Information included in the manuscript?	In which section is the information available? (Reagents and Tools Table, Materials and Methods, Figures, Data Availability Section)
Studies involving **human participants**: State details of **authority granting ethics approval** (IRB or equivalent committee(s), provide reference number for approval.	Not Applicable	
Studies involving **human participants**: Include a statement confirming that **informed consent** was obtained from all subjects and that the experiments conformed to the principles set out in the WMA Declaration of Helsinki and the Department of Health and Human Services Belmont Report.	Not Applicable	
Studies involving **human participants:** For publication of **patient photos**, include a statement confirming that consent to publish was obtained.	Not Applicable	
Studies involving experimental **animals**: State details of **authority granting ethics approva**l (IRB or equivalent committee(s), provide reference number for approval. Include a statement of compliance with ethical regulations.	Yes	All animal procedures were approved by the Government of Upper Bavaria (animal protocol 55.2-1-54-2532-135-15), and all the methods were performed in accordance with the relevant guidelines and regulations.
Studies involving **specimen and field samples:** State if relevant **permits** obtained, provide details of authority approving study; if none were required, explain why.	Not Applicable	

Dual Use Research of Concern (DURC)	Information included in the manuscript?	In which section is the information available? (Reagents and Tools Table, Materials and Methods, Figures, Data Availability Section)
Could your study fall under dual use research restrictions? Please check biosecurity documents and list of **select agents and toxins** (CDC): https://www.selectagents.gov/sat/list.htm	Not Applicable	
If you used a select agent, is the security level of the lab appropriate and reported in the manuscript?	Not Applicable	
If a study is subject to dual use research of concern regulations, is the name of the **authority granting approval and reference number** for the regulatory approval provided in the manuscript?	Not Applicable	

Reporting

The MDAR framework recommends adoption of discipline-specific guidelines, established and endorsed through community initiatives. Journals have their own policy about requiring specific guidelines and recommendations to complement MDAR.

Adherence to community standards	Information included in the manuscript?	In which section is the information available? (Reagents and Tools Table, Materials and Methods, Figures, Data Availability Section)
State if relevant guidelines or checklists (e.g., **ICMJE, MIBBI, ARRIVE, PRISMA**) have been followed or provided.	Yes	We followed the ARRIVE guidelines wherever possible.
For **tumor marker prognostic studies,** we recommend that you follow the **REMARK** reporting guidelines (see link list at top right). See author guidelines, under 'Reporting Guidelines'. Please confirm you have followed these guidelines.	Not Applicable	
For **phase II and III randomized controlled trials**, please refer to the **CONSORT** flow diagram (see link list at top right) and submit the CONSORT checklist (see link list at top right) with your submission. See author guidelines, under 'Reporting Guidelines'. Please confirm you have submitted this list.	Not Applicable	

Data Availability

Data availability	Information included in the manuscript?	In which section is the information available? (Reagents and Tools Table, Materials and Methods, Figures, Data Availability Section)
Have **primary datasets** been deposited according to the journal's guidelines (see 'Data Deposition' section) and the respective accession numbers provided in the Data Availability Section?	Yes	This study includes no data deposited in external repositories
Were **human clinical and genomic datasets** deposited in a public access-controlled repository in accordance to ethical obligations to the patients and to the applicable consent agreement?	Not Applicable	
Are **computational models** that are central and integral to a study available without restrictions in a machine-readable form? Were the relevant accession numbers or links provided?	Not Applicable	
If publicly available data were reused, provide the respective **data citations in the reference list**.	Not Applicable	
